# Enrichment and structural assignment of geometric isomers of unsaturated furan fatty acids

**DOI:** 10.1007/s00216-023-04908-z

**Published:** 2023-08-21

**Authors:** Franziska Müller, Jürgen Conrad, Tim Hammerschick, Walter Vetter

**Affiliations:** 1 Department of Food Chemistry (170b), Institute of Food Chemistry, University of Hohenheim, Garbenstr. 28, Stuttgart, 70599 Germany; 2 Department of Bioorganic Chemistry (130b), Institute of Chemistry, University of Hohenheim, Garbenstr. 30, Stuttgart, 70599 Germany

**Keywords:** Unsaturated furan fatty acid, Latex, Countercurrent chromatography, Silver ion chromatography, NMR

## Abstract

Furan fatty acids (FuFAs) are valuable minor fatty acids, which are known for their excellent radical scavenging properties. Typically, the furan moiety is embedded in an otherwise saturated carboxyalkyl chain. Occasionally, these classic FuFAs are accompanied by low amounts of unsaturated furan fatty acids (uFuFAs), which additionally feature one double bond in conjugation with the furan moiety. A recent study produced evidence for the occurrence of two pairs of *E*-/*Z*-uFuFA isomers structurally related to saturated uFuFAs. Here, we present a strategy that allowed such trace compounds to be enriched to a level suited for structure determination by NMR. Given the low amounts and the varied abundance ratio of the four uFuFA isomers, the isolation of individual compounds was not pursued. Instead, the entire isomer mixture was enriched to an amount and purity suitable for structure investigation with contemporary NMR methods. Specifically, lipid extracted from 150 g latex, the richest known source of FuFAs, was subsequently fractionated by countercurrent chromatography (CCC), silver ion, and silica gel column chromatography. Analysis of the resulting mixture of four uFuFAs isomers (2.4 mg in an abundance ratio of 56:23:11:9) by different NMR techniques including PSYCHE verified that the structures of the two most abundant isomers were *E*-9-(3-methyl-5-pentylfuran-2-yl)non-8-enoic acid and *E*-9-(3-methyl-5-pent-1-enylfuran-2-yl)nonanoic acid. Additionally, we introduced a computer-based method to generate an averaged chromatogram from freely selectable GC/MS runs of CCC fractions without the necessity of pooling aliquots. This method was found to be suitable to simplify subsequent enrichment steps.

**Graphical Abstract:**

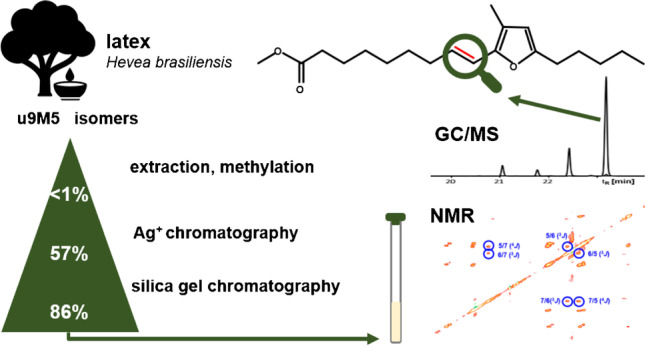

**Supplementary information:**

The online version contains supplementary material available at 10.1007/s00216-023-04908-z.

## Introduction

**Fig. 1:**
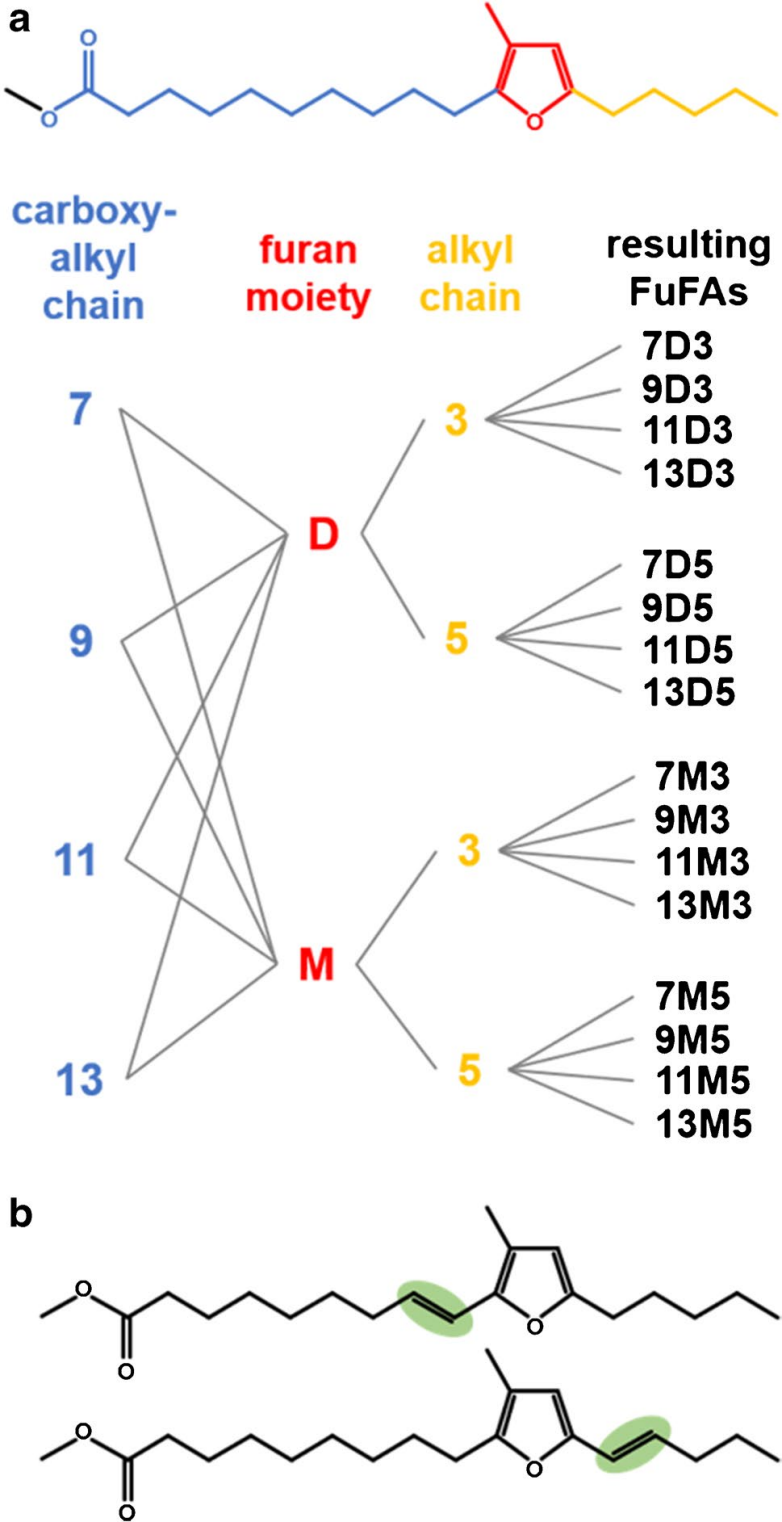
**a** Structure of 9-(3-methyl-5-pentylfuran-2-yl)nonanoic acid methyl ester (9M5-ME) and a scheme with all combinations of the most common substitution patterns of three subunits of furan fatty acids (FuFAs), i.e., a carboxyalkyl chain with 7, 9, 11, or 13 carbon atoms, a β-mono- or β,βʹ-dimethylated furan moiety (M-FuFA/D-FuFA), and either a propyl or a pentyl residue. Individual FuFAs, shown on the right side, were abbreviated according to Vetter et al. [7]. **b** Structure of two *E*-configured unsaturated furan fatty acids (uFuFAs), i.e., *E*-9-(3-methyl-5-pentylfuran-2-yl)-non-8-enoic acid methyl ester (9:1M5-ME) and *E*-9-(3-methyl-5-pent-1-enylfuran-2-yl)-nonanoic acid methyl ester (9M5:1-ME) [8]

Furan fatty acids (FuFAs) are minor constituents of the lipid fraction of various biological samples and foods which are valued due to their prodigious free radical scavenging and antioxidative properties [1–5]. Structurally, the eponymous furan moiety is α-connected with a carboxyalkyl chain (mostly consisting of 7, 9, 11, or 13 carbon atoms) and αʹ-connected with either a propyl or a pentyl residue [3, 6]. In addition, the furan moiety of naturally occuring FuFAs features either one methyl group in β-position (family of M-FuFAs) or two methyl groups in β,βʹ-positions (family of D-FuFAs) [3]. The combination of the most common variation pattern of the three subunits — carboxyalkyl chain (*n* = 4), FuFA family (*n* = 2), alkyl chain (*n* = 2) — gives rise to (4 × 2 × 2 =) 16 FuFAs most frequently detected in biological samples (Fig. 1a). Complex chemical names of FuFAs have led to the introduction of “number–letter–number” short-terms which allow the structure to be recognized directly [7]. Specifically, the first number denotes the length of the carboxyalkyl chain, the subsequent letter indicates the FuFA family (where M represents a M-FuFA and D a D-FuFA), and the final number states the length of the alkyl chain (Fig. 1a) [7]. Hence, the FuFA 9-(3-methyl-5-pentylfuran-2-yl)nonanoic acid is termed 9M5.

Occasionally, the predominant chain-saturated FuFAs were accompanied by very low amounts of two structurally related unsaturated FuFAs (uFuFAs) — e.g., unsaturated 11D5 (u11D5) isomers. GC/MS evaluation indicated that these isomers bear an additional double bond in conjugation with the furan moiety either in the carboxyalkyl chain (11:1D5) or the alkyl chain (11D5:1) (Fig. 1b) [9–12]. Moreover, Jandke et al. (1988) [13] and recently Müller et al. (2023) [8] not only detected two but four uFuFA isomers, which produced strong evidence for the existence of *Z*- and *E*-isomers [8, 13]. Noteworthy and valid for five isomeric groups of uFuFA (u9D5 [13], u11D3, u11D5, u11M5, and u9M5 [8]), two isomers were predominant, and two were only low abundant, which might explain why two of the isomers had been overlooked in most of the previous studies that reported the presence of uFuFAs in fish (oil) [8, 10, 14, 15], beef liver [16], olive oil [11], or herbs [12], and in laboratory studies [13, 16]. However, the final structural proof of uFuFA isomers had not been achieved. Unfortunately, multi-step syntheses of (chain-saturated) FuFAs [17, 18] are difficult to conduct, and paired with additional double bonds and the geometry issue of uFuFAs, it appeared unrealistic to be feasible in reasonable time. Vice versa, knowledge of the exact uFuFA structures would simplify their later synthesis by experts. Therefore, the main focus was laid on the enrichment of the uFuFAs from a sample comparably rich in FuFAs to a degree that enabled the first structure determination by NMR.

Until today, only one natural source rich in FuFAs has been discovered, i.e., *Hevea brasiliensis* [19, 20]. The latex of this tree contained 9M5 at up to 76% of the total fatty acids depending on the genotype [21, 22]. Later, it was found that 9M5 was also high abundant in laboratory latex gloves made from *Hevea brasiliensis* from which it could be isolated in mg amounts [23]. More recently, it was found that this matrix also contained traces of uFuFAs that were structurally related to 9M5 (u9M5 isomers) [8]. However, the obtained uFuFA amounts were too small and the purity too poor for a subsequent structure elucidation by NMR. In this context, it was reasonable to assume that the existing NMR data on 9M5 [19, 21, 23, 24] would simplify the structural assignment of u9M5 isomers.

In this study, we thus aimed to enrich u9M5 isomers from latex (gloves) in amounts suitable for subsequent structure elucidation by NMR methods. Given the low proportion of u9M5 next to 9M5, the application of countercurrent chromatography (CCC) was deemed best suited for the enrichment of u9M5 isomers. CCC is an all-liquid–liquid chromatography method valued for the isolation and enrichment of (low concentrated) natural products [25–27] including FuFAs [24, 28, 29]. The setup of CCC instruments is similar to HPLC systems except that the HPLC column is substituted with the so-called CCC centrifuge. By means of centrifugal forces, one phase of the biphasic solvent system is kept stationary, while the other one serves as the mobile phase. The elution time of an analyte is directed by its partition coefficient in the biphasic solvent system. Nevertheless, depending on the composition of the sample, co-elutions are frequently inevitable in CCC. Hence, a complementary technique — silver ion column chromatography — was required. Prior to NMR investigation, the remaining impurities were removed from the mixture of four u9M5 isomers by means of a specifically modified column chromatography method.

## Materials and methods

### Samples, analytical standards, and chemicals

Disposable latex gloves (powder free, size S; 100 gloves per unit) were obtained from vwr (Darmstadt, Germany). Myristic acid (14:0, purity > 98%, Fluka, Steinheim, Germany) was ethylated as described elsewhere [30] and used as internal standard (IS) to level out run-to-run variations of the GC/MS system [31]. A fatty acid methyl ester 37 component FAME mix reference standard was purchased from Sigma-Aldrich, Steinheim, Germany (Table S1). The transesterified FuFA standards 9M5-ME (purity > 98%, determined by GC/MS) and 9D5-ME (purity > 95% determined by GC/MS) were isolated and purified from disposable latex gloves [32] or from wild grown meadow mushrooms [31], respectively. A well-studied and FuFA-containing fish oil (PronovaPure 360:240 TG) was provided by BASF (Ludwigshafen am Rhein, Germany) [2].

Ethyl acetate, ethanol, methanol, and* n*-hexane (all HPLC grade) were ordered from Th. Geyer (Renningen, Germany). Diethyl ether (≥ 99.5% for analysis p.a.; stabilized with ~ 7 ppm 2,6-di-*tert*-butyl-4-methylphenol (BHT); low in peroxide) and acetonitrile (ACN) were from Bernd Kraft (Duisburg, Germany). Deuterated chloroform (CDCl_3_, ≥ 99.8%) was obtained from Deutero (Kastellaun, Germany). Silica gel 60, sodium chloride (≥ 98.5%), sodium sulfate (≥ 99%), and silver nitrate (≥ 99.5%) were purchased from Sigma-Aldrich (Steinheim, Germany). Concentrated hydrochloric acid (32%) and concentrated sulfuric acid (96%, for analysis p.a.) were obtained from Carl Roth (Karlsruhe, Germany).

### Extraction and transesterification of fatty acids from latex

Thirty-six disposable latex gloves (150 g) were cut to small pieces, and three portions of 50 g of each were extracted three times with 900 mL *n-*hexane, respectively. The *n-*hexane extracts were combined, evaporated, transesterified with 1% sulfuric acid in methanol for 4 h, and the formed FAMEs were extracted with *n-*hexane, dried with sodium sulfate, and concentrated on the rotary evaporator [8].

### Enrichment of u9M5-ME by countercurrent chromatography (CCC)

u9M5 methyl ester (u9M5-ME) isomers were enriched using coils 2 + 3 (total volume 236 mL) of a QuikPrep MK8 system with the setup described in detail by Hammann et al. (2015) [33]. The FAME solution was separated in tail-to-head mode (upper phase = mobile phase) with the solvent system *n*-hexane/acetonitrile (1:1; v:v) [29], which showed a stationary phase retention (S_f_) of 88% at a mobile flow rate of 2 mL/min. The effluent was monitored with a flash 10 diode array detector (DAD, Ecom Praha, Czech Republic) which was set at λ_1_ = 230 nm, λ_2_ = 260 nm, and λ_3_ = 290 nm. Between 42 to 202 mL after the injection, 40 fractions (CCC fractions 1–40) of 4 mL each were taken and evaporated to dryness by a gentle stream of nitrogen (38 °C). Fraction weights were determined gravimetrically, and the residues were dissolved in 1 mL *n*-hexane each. An aliquot of each fraction was appropriately diluted, supplemented with 4 µg 14:0-EE as IS, and measured with GC/MS both in the scan and the selected ion monitoring (SIM) mode. The GC/MS data were evaluated, and the elution profile was created according to Müller et al. (2019) [34]. The partition coefficients (*K* value), showing the substance-specific distribution between the two immiscible phases used in CCC separation, were calculated for all identified FAMEs using the fraction data of the CCC fraction with the highest abundance for each substance, as reported elsewhere [25, 31].

### Fractionation of u9M5-ME containing CCC fractions by silver ion chromatography 

Silver ions (Ag^+^) coated on silica gel form charge–transfer complexes with double bonds of the analytes, and therefore, this method basically separates FAMEs according to the number, configuration, and partly to the position of double bonds [35]. In the present study (see previous section), CCC fractions 25–37 were pooled, concentrated to ~ 1 mL, and subjected silver ion chromatography (5 g of 1% deactivated silver nitrate silica gel (20% AgNO_3_, iron-free)) according to Müller et al. (2023) [8] with the following modifications: (i) Ag^+^-fraction I (*n*-hexane/diethyl ether, 99.5:0.5, v/v; targeted compounds: saturated FAMEs) was omitted, and the fractionation started with Ag^+^-fraction II (*n*-hexane/diethyl ether, 97:3, v/v, 70 mL, target compounds: saturated FuFA-ME) on a preconditioned column using Ag^+^-eluent II. (ii) Ag^+^-eluent III consisted of 50 mL *n*-hexane/ethyl acetate, 80:20, v/v (instead of *n*-hexane/diethyl ether, 80:20, v/v, target compounds: u9M5 isomers). Namely, diethyl ether was substituted due to its required stabilization with BHT to prevent the formation of peroxides [31]. Yet, the presence of BHT would have been detrimental for NMR measurements. Both Ag^+^-fractions were concentrated using a rotary evaporator, transferred in a pre-weighted vial, and the solvent was removed under a gentle stream of nitrogen (38 °C). After the residue was weighted, dissolved in 1 mL *n*-hexane, suitable diluted (final volume 0.1 mL), and supplemented with 0.4 µg 14:0-EE as IS, the Ag^+^-fractions were analyzed by GC/MS in full scan mode.

### Final enrichment of u9M5 isomers by silica gel column chromatography 

Ag^+^-fraction III (see previous section) was further purified according to Krauss et al. (2016) [36] with slight changes regarding the eluent II and the stationary phase. A glass column (diameter 1 cm) was packed with 5 g 20% deactivated iron-free silica gel [31]. After conditioning with *n*-hexane, Ag^+^-fraction III was applied, and silica fraction I was eluted with 30 mL *n*-hexane, followed by four silica subfractions (IIA–IID) of 10 mL *n*-hexane/ethyl acetate, 98:2, v/v; 40 mL. Silica subfractions IIA–IID were individually evaporated (38 °C, N_2_), weighted, and re-dissolved in 1 mL *n*-hexane. Aliquots (1–10 µL) of each silica subfraction were diluted according to their weight (final volume 0.1 mL), spiked with 4 µL IS solution (featuring 0.4 µg 14:0-EE), and analyzed by GC/MS in full scan mode. The remaining share of the silica subfraction IIC not used for measurement was evaporated, taken up in 700 µL CDCl_3_, and applied to NMR analysis.

### Gas chromatography with mass spectrometry (GC/MS)

Gas chromatography with mass spectrometry (GC/MS) was used for the analysis of methyl esters of conventional fatty acids (FAMEs), FuFAs, and uFuFAs present in CCC, silver ion, and silica fractions. Measurements were performed with an HP 6890 GC plus/5973N MSD instrument (Agilent, Waldbronn, Germany) equipped with an Rtx-2330 capillary column (90% biscyanopropyl, 10% cyanopropylphenyl polysiloxane, 60 m × 0.25 mm internal diameter × 0.1 µm film thickness, Restek Bellefonte, PA, USA). Samples were injected via an automatic liquid sampler (6890 ALS) system (Agilent, Waldbronn, Germany) and measured in splitless mode using the settings described elsewhere [32]. GC/MS spectra were recorded in the full scan mode (*m/z* 50–650) after a solvent delay of 7 min [32].

Conventional FAMEs were identified by means of a 37-component FAME mix reference standard (section “Samples, analytical standards, and chemicals”) by the relative retention time (RRI) based on the first and last eluting conventional FAME in the latex extract (12:0-ME and 18:3*n*-3-ME). RRI ($RRI= \frac{{t}_{R, Analyt}}{({t}_{R, 12:0-ME}+{t}_{R, 18:3n-3-ME})}\bullet 1.5$) was calculated according to Vetter et al. (1997) [37] (Table S1 and S2).

Next to the retention times, FAME structures were verified by means of the molecular ion (M^+^) along with the abundance ratios of the characteristic ions *m/z* 74, 79, 81, and 87. In addition, *n-3*- and *n-6*-polyunsaturated fatty acid methyl esters (PUFA-ME) were assigned via the relative abundance of *m/z* 108 and *m/z* 150, respectively (Table S1) [38, 39]. FuFA-MEs were unequivocally identified by M^+^ and four characteristic fragment ions in the GC/MS spectra as reported elsewhere (Table S2) [10, 31, 40]. GC/MS retention time of reference standards (9M5-ME, 9D5-ME) and FuFAs present in a well-studied fish oil [2] (section “Samples, analytical standards, and chemicals”) were used for verification. uFuFA-MEs were identified by their GC/MS spectra (Table S2) and their elution pattern according to Müller et al. (2023) [8]. In this context, it is remarkable that GC/MS data of uFuFA-ME were not included in the online database [41].

### Creation of an averaged GC/MS chromatogram for selected CCC fractions

For the selection of the most suitable CCC fraction used in further enrichment and cleanup steps, averaged GC/MS chromatograms of CCC fractions 25–37 (or less fractions) were created by exporting the data points (148 data points per minute) of the thirteen GC/MS chromatograms to Microsoft Excel 2019 (Microsoft, Redmond, WA, USA). The abundance data (*y* axis parameter) of each chromatogram was first normalized with the IS (14:0-EE) to level out run-to-run variations. Specifically, the area of the IS was determined in each fraction using MSD ChemStation E. 02.00.493 (Agilent, Waldbronn, Germany), and a correction factor CF1 was calculated for each fraction (${\mathrm{CF}1}_{i}=\frac{\overline{{\mathrm{A} }_{\mathrm{IS}}}}{{\mathrm{A}}_{{\mathrm{IS}}_{i}}}$ with ${\mathrm{A}}_{{\mathrm{IS}}_{i}}$ as the peak area of IS in the *i*^th^ CCC fraction and $\overline{{\mathrm{A} }_{\mathrm{IS}}}$ as the mean of the peak area of IS in all selected samples). In the next step, all data points (y axis value) from each fraction were multiplied with its determined CF1 and its dilution factor (DF). Then, the corrected abundance data points of the thirteen chromatograms were added for each retention time data point (x value). Afterwards, the sum was divided by the mean dilution factor ($\overline{\mathrm{DF} }$) of the thirteen fractions, and the final *y* axis values were plotted against the retention time (x axis value).

### NMR measurements

NMR spectra were measured in CDCl_3_ on a Bruker AVANCE III HD 600 spectrometer with a 5 mm BBO Prodigy cryo-probe (Bruker, Billerica, MA, USA). Chemical shifts were referenced to residual solvent signals of CDCl_3_ at δ = 7.26 ppm for ^1^H and 77.0 ppm for ^13^C. Correlated spectroscopy (COSY), heteronuclear single quantum coherence (HSQC), heteronuclear multiple bond correlation (HMBC), and 1D selected total correlation spectroscopy (selTOCSY) spectra were recorded with standard 1D and 2D Bruker pulse sequences within TopSpin 3.6.1 (copyright 2018, Bruker Biospin, Billerica, MA, USA). A super long-range HMBC was recorded by an in-house modified Bruker pulse sequence [42, 43]. Triple spin echo pure shift yielded by chirp excitation (TSE-PSYCHE) and F1-homodecoupled PSYCHE TOCSY parameters were obtained from the Manchester NMR methodology group [44–46]. The F1-homodecoupled clean-in-phase (CLIP) COSY experiment was implemented from the online BRUKER user library [47–49]. NMR spectra were processed with Topspin 4.1.3 (copyright 2021, Bruker Biospin, Billerica, MA, USA) and SpinWorks 4.2.10 (Copyright 2019, K. Marat, University of Manitoba, CA).

## Results and discussion

### Enrichment of u9M5-ME isomers from latex

**Fig. 2:**
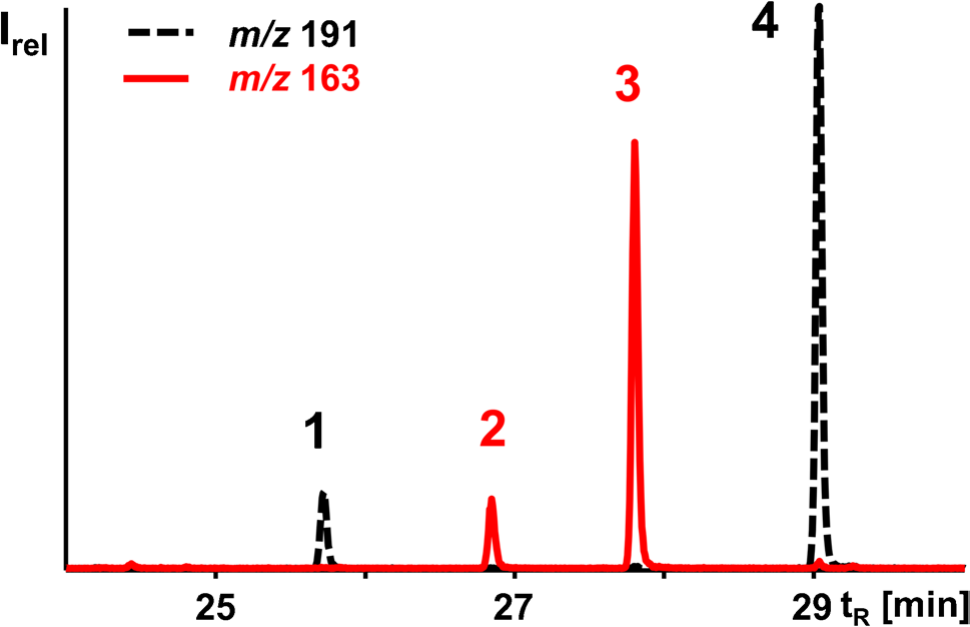
GC/MS ion chromatograms of the base peaks of 9:1M5-ME (*m/z* 191) and 9M5:1-ME (*m/z* 163) isomers extracted from GC/MS analysis of CCC fraction 31. The corresponding full scan spectra are shown in Fig. S1

Fatty acids in the *n*-hexane extract of 150 g disposable latex gloves were converted into fatty acid methyl esters (FAMEs). The whole transesterified sample obtained in this way (~ 1 g FAMEs) was fractionated in one CCC run in tail-to-head mode. Forty CCC fractions were taken, and the four u9M5-ME isomers were detected in CCC fractions 25–40 with the highest abundance in CCC fraction 31 (K value 0.64) according to GC/MS analysis. Specifically, the diagnostic base peak in the GC/MS spectra verified the presence of two u9M5 isomers with an additional double bond in the carboxylalkyl chain (9:1M5-ME, *m/z* 191, peaks 1 and 4) and two isomers with a double bond in the pentyl chain (9M5:1-ME, *m/z* 163, peaks 2 and 3; Fig. 2, Table S2–S3) [8].

**Fig. 3:**
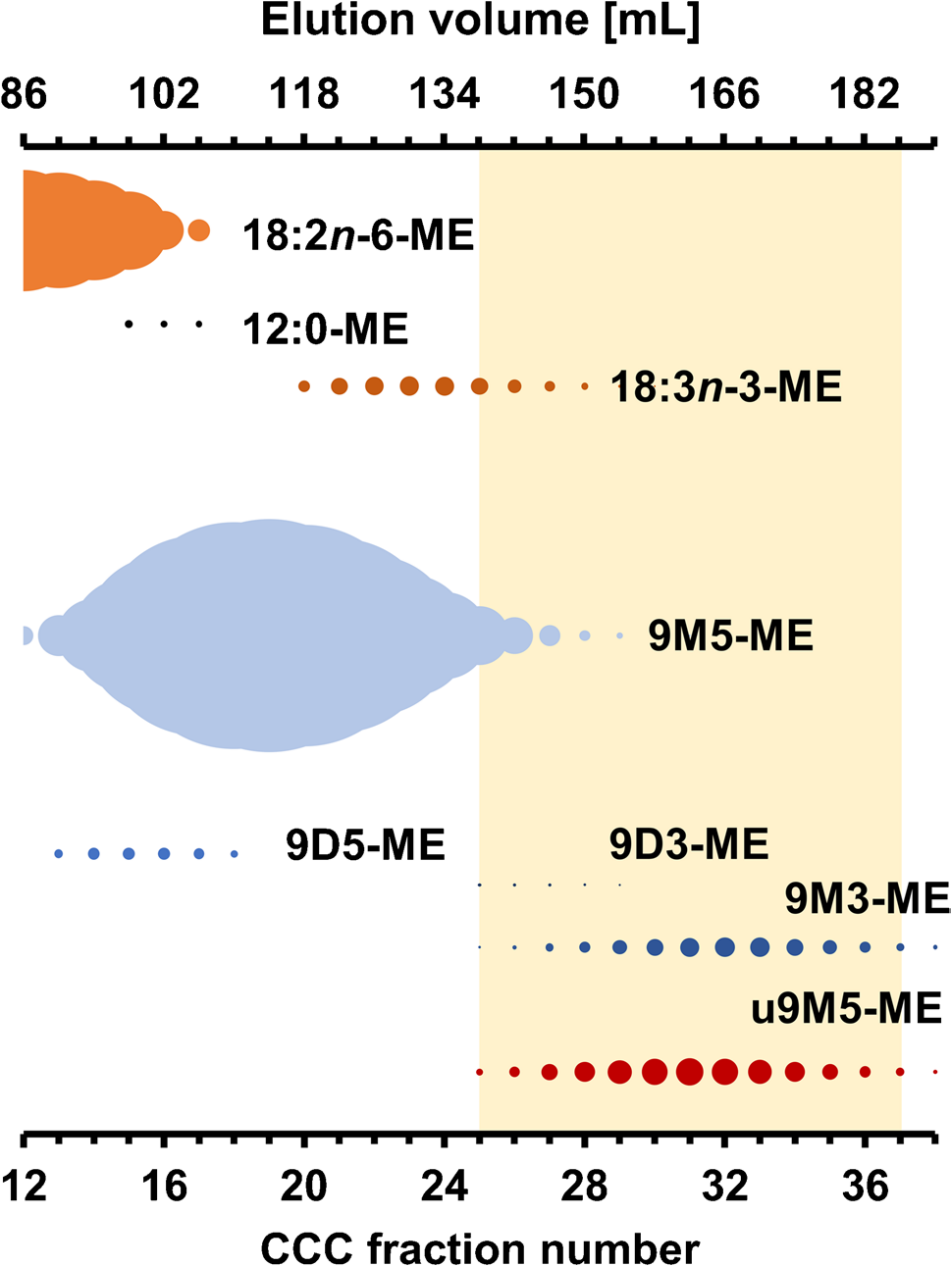
Bubble plot of the elution profile of different fatty acid methyl esters (FAMEs) including the analytes (uFuFA-ME) plotted against the fraction number and the elution volume [mL]. The sizes of the bubbles indicate the amount of each compound in each fraction. Before the plotting, the data were smoothed according to Müller et al.(2019) [34]

The isomer pattern of the methyl esters of the uFuFAs (uFuFA-ME) was virtually identical in CCC fractions 25–37 (K value range 0.52–0.75), and the different isomers were not even partially separated (Fig. S2). In tail-to-head mode (nonpolar, upper phase = mobile phase), the bulk of the earlier eluting FuFA 9M5-ME (~ 68% of total FAMEs) could be removed from the elution range of the uFuFAs (Fig. 3, Fig. S2).

Similarly, saturated FAMEs, linoleic acid methyl ester (18:2*n*-6-ME; CCC fraction 8–17), and the bulk of α-linolenic acid methyl ester (18:3*n*-3-ME; CCC fractions 20–30) could be removed with this step. Next to low shares of 9M5-ME and 18:3*n*-3-ME, the elution range of the u9M5-ME isomers fully overlapped with the one of 9D3-ME (CCC fractions 25–29, ~ 0.1% of u9M5-ME isomers) and 9M3-ME (CCC fractions 25–39, ~ 53% of u9M5-ME isomers). Both 9D3-ME and 9M3-ME were hardly or even not visible in GC/MS chromatogram before the CCC separation, but they decisively reduced the purity of u9M5-ME isomers in CCC fractions 25–37 (Fig. 3, Fig. S2). This co-elution can also be seen in the very similar *K* values of these substances (9D3: 0.54, 9M3: 0.66; Table S2).

According to the equivalent chain length (ECL) rule for conventional fatty acids in CCC (ECL = *n*_c_ − 2 × *n*_d_ with *n*_c_ number of carbon atoms and *n*_d_ number of double bonds), the co-elution of 9M3-ME and u9M5-ME isomers was difficult to overcome by CCC which necessitated the application of an alternative separation method. However, the CCC step decisively reduced the sample weight from initially ~ 1 g to merely 20.1 mg in pooled CCC fractions 25–37. This considerably low sample weight after the CCC step was suited for the application of analytical methods with lower sample capacity.

**Fig. 4:**
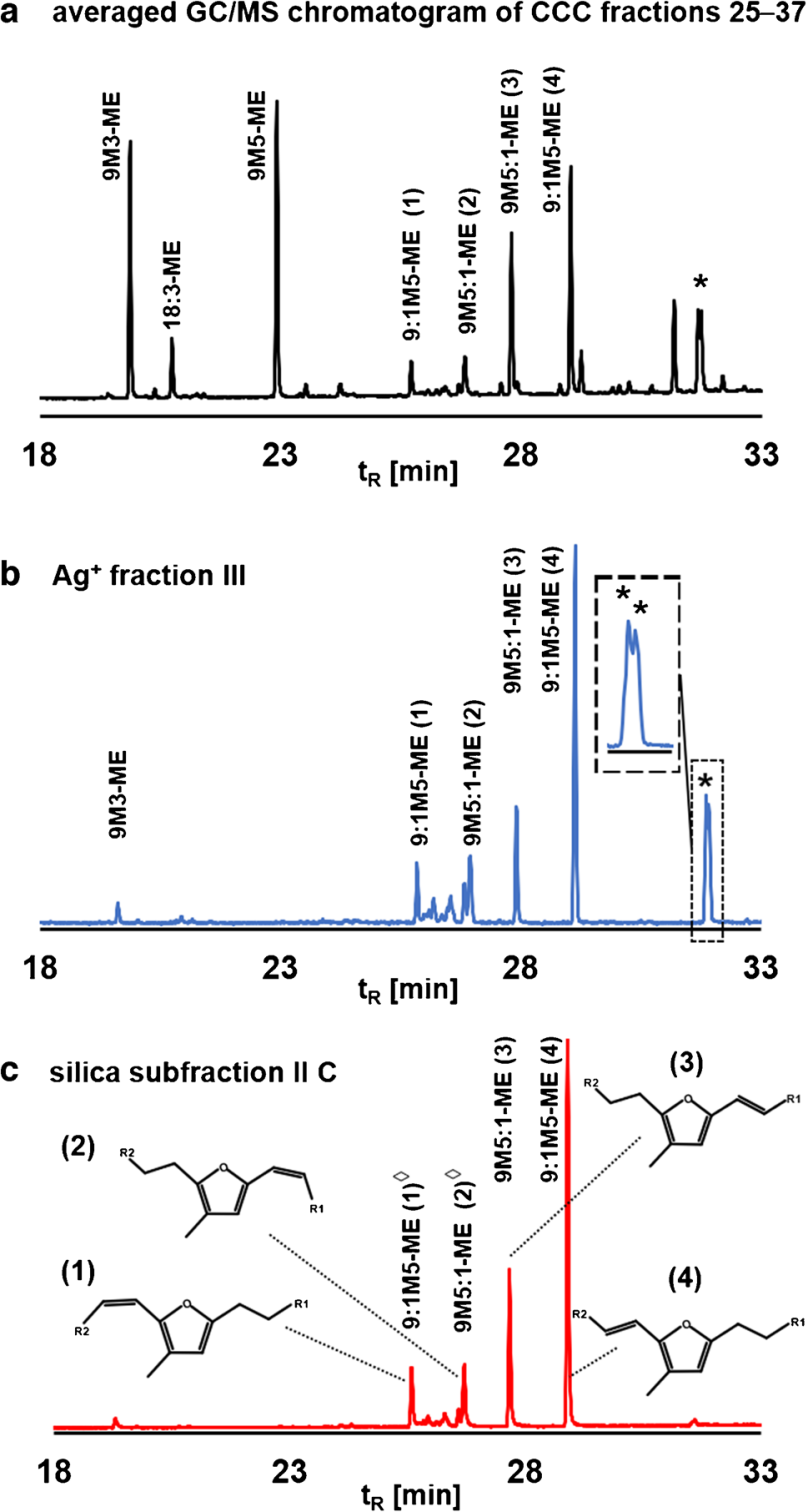
Overview of the cleanup efficiency in the individual steps: **a** averaged GC/MS chromatogram (section “Creation of an averaged GC/MS chromatogram for selected CCC fractions”) of CCC fractions 25–37 (excerpt), **b** GC/MS chromatogram of silver ion fraction III of CCC fractions 25–37 (excerpt). **c** GC/MS chromatogram of silica subfraction IIC which was used for the NMR measurement (excerpt). Structures of isomers marked with a diamond ^◊^ were not determined with NMR. Inserted are the structures of the four u9M5-ME isomers with *R*_1_ = (CH_2_–CH_3_) and *R*_2_ = ((CH_2_)_5_–COO–CH_3_)

In order to select the best suited CCC fractions for a subsequent separation step, we are introducing a new illustration approach. Namely, the thirteen GC/MS chromatograms of CCC fraction 25–37 were individually normalized (section “Creation of an averaged GC/MS chromatogram for selected CCC fractions”). Merging of the individual x  (retention time) and y (normalized abundance) data point of the thirteen GC/MS runs resulted in the averaged GC/MS chromatogram of CCC fractions 25–37 without the cumbersome necessity of the exact pooling of aliquots of each CCC fraction. This mean GC/MS chromatogram of CCC fraction 25–37 indicated that the two most abundant u9M5-ME isomers were among the five most prominent compounds in this elution range (Fig. 4a). With this new approach, freely selectable CCC fractions can be included or excluded in order to simulate how this influences the amount and purity of the u9M5-ME isomers in the pooled sample. In this way, it was found out that only the omission of CCC fraction 25 would have slightly improved the purity without losing decisive amounts of u9M5-ME isomers.

 Since the major impurities 9M3-ME, 9M5-ME, and 18:3*n*-3-ME differed in number or relative position of double bonds from the uFuFA-ME isomers (isolated double bonds in the case of 18:3*n*-3-ME and a double bond in conjugation with a furan moiety in u9M5-MEs), a further purification step was carried out by silver ion chromatography. As silver ion chromatography was suited for the separation of all mentioned FAMEs from the u9M5-MEs, CCC fraction 25–37 were pooled for this step, in order to keep the yield as high as possible.

A slightly modified elution scheme (including substitution of diethyl ether with ethyl acetate) facilitated the removal of the saturated FuFA-ME 9M5-ME and 9M3-ME (silver ion fraction II) [12] from u9M5-ME isomers (silver ion fraction III) [12] and also from 18:3*n*-3-ME, which remained on the column (Fig. 4a, b). Silver ion fraction II was not further investigated because NMR data of saturated FuFAs has been reported several times [19, 21, 23, 24]. However, the presence of two remaining impurities in silver ion fraction III (Fig. 4b, peaks marked with an asterisk) necessitated the implementation of a further cleanup step on silica gel before the application of NMR.

Using this protocol, both interfering compounds eluted only slightly earlier than the four u9M5-ME isomers into silica fraction II (Fig. S3). Silica subfraction IIC showed both the highest amount (2.4 mg) and purity (86%, determined by GC/MS) of the four u9M5-ME according to GC/MS (Fig. 4c). The share of the individual u9M5-ME isomers in this fraction was 9% peak 1 (9:1M5-ME isomer), 12% peak 2 (9M5:1-ME isomer), 23% peak 3 (9M5:1-ME isomer), and 56% peak 4 (9:1M5-ME isomer). Finally, this tremendous liquid chromatographic enrichment protocol (CCC, silver ion, and silica chromatography), supervised by GC/MS measurements, was rewarded by obtaining a fraction with four u9M5-ME isomers which was suited for NMR investigation.

### Structure elucidation of u9M5-ME isomers by NMR

**Fig. 5:**
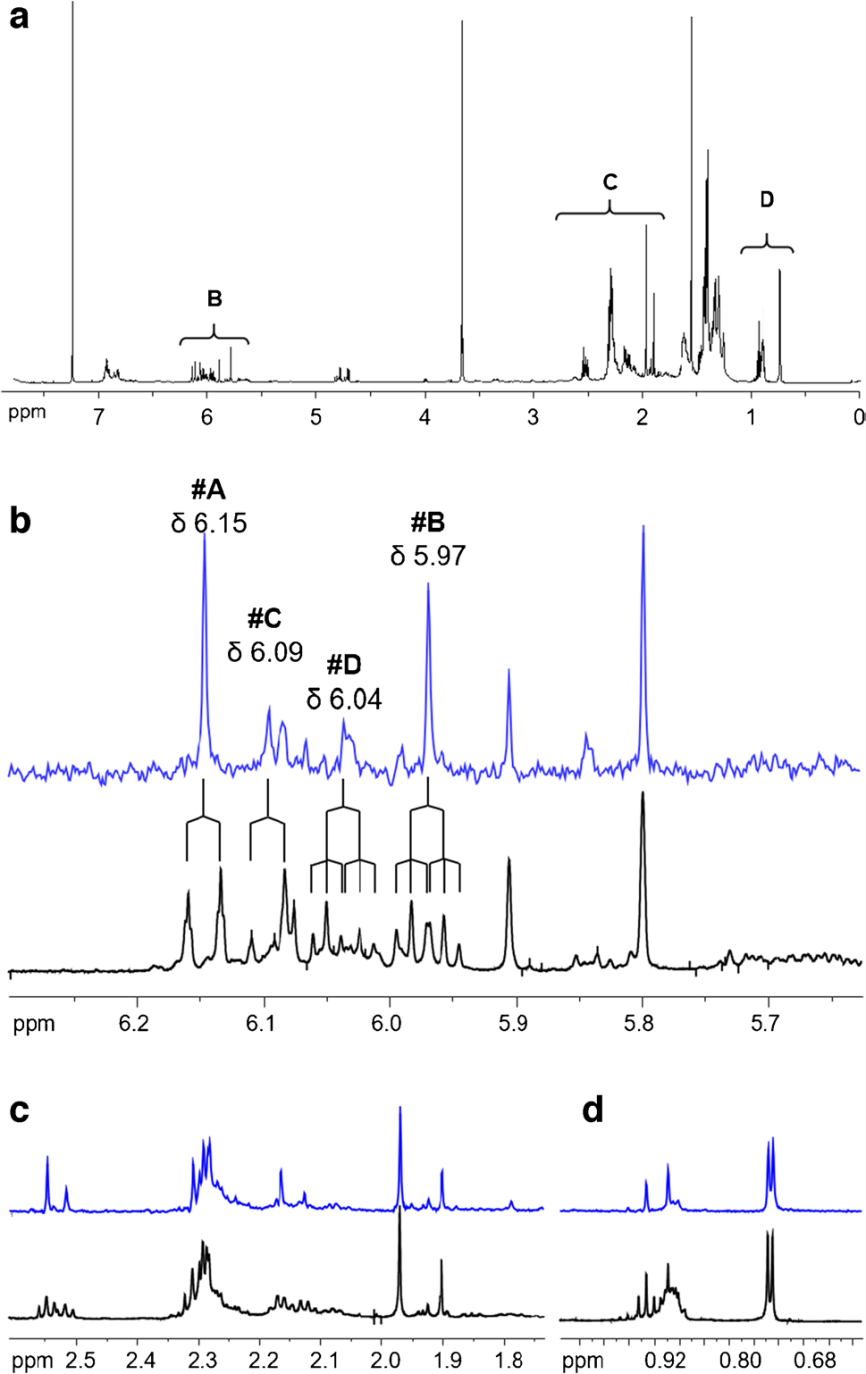
^1^H NMR spectrum (600 MHz, CDCl_3_) of subfraction silica IIC in CDCl_3_ (**a**). Enlarged excerpts (**b**–**d**) display expansions of the ^1^H NMR (black) and fully homoband decoupled ^1^H NMR by PSYCHE experiment (blue)

The ^1^H NMR spectrum of silica subfraction IIC showed various poorly resolved signals which were difficult to interpret (Fig. 5a). Yet, enlargements of specific ppm ranges verified that the chemical shifts were in the anticipated range which was known from NMR data of 9M5-ME (Fig. 5, black track) [21, 24].

Signal assignments were simplified by recording a broadband homonuclear decoupled ^1^H NMR spectrum based on PSYCHE [44–46], displaying only singlets at the respective chemical shifts (Fig. 5b–d, blue track). The resulting gain in information was crucial in the present case due to the presence of several compounds in a mixture. Extensive 2D NMR analysis including broadband homonuclear decoupled 2D NMR techniques based on PSYCHE decoupling schemes [44–49] enabled the unequivocal assignment of the signals of the two most abundant u9M5-ME isomers. Namely, the ^1^H NMR spectrum of the major isomer 9:1M5-ME (peak 4, Fig. 4c) featured two diagnostic olefinic protons at δ 5.97 ppm (H8, dt, *J*_*1*_ = 15.6 Hz, *J*_*2*_ = 7.0 Hz) and at δ 6.15 ppm (H9, d, *J* = 15.7 Hz) (Fig. 5b, signals **#**A and #B).

**Fig. 6:**
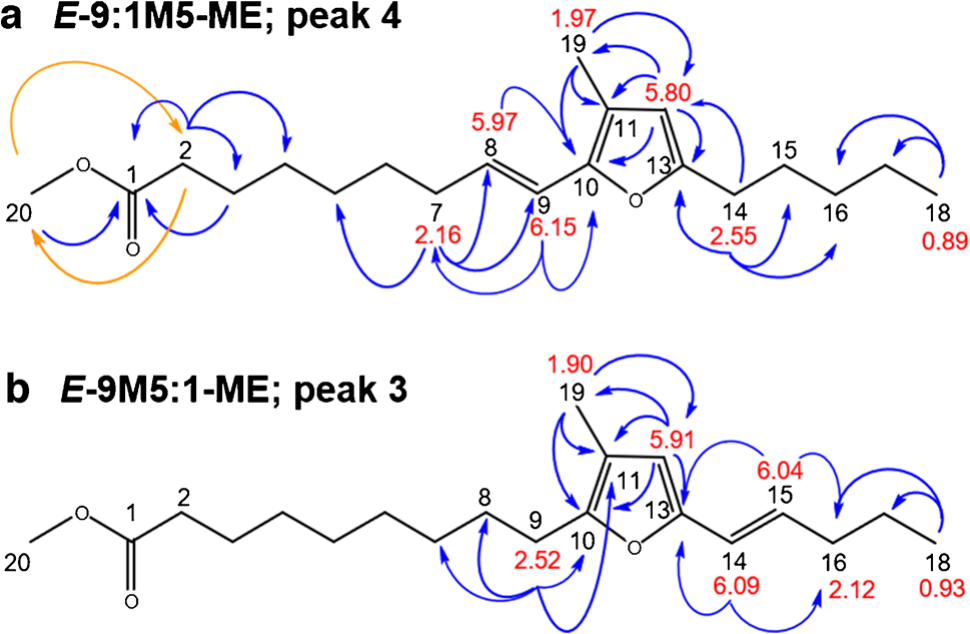
Chemical structures of the two u9M5-ME isomers identified in this study. Key HMBC correlations are shown in blue color and super long-range HMBC in orange color. Important chemical shifts (δ_H_ [ppm]) are inserted in red color

Both the coupling pattern, the size of the coupling constants of H8, and their ^13^C chemical shifts (C8: δ 126.8 ppm and C9: δ 117.0 ppm) consecutively agreed with an *E*-configurated double bond vicinal to a methylene group (H7 via H8), while H9 was adjacent to the quaternary carbon C10 of the furan moiety (no additional coupling, Fig. 6a).

COSY correlations between H8 and the aforementioned methylene protons H7 at δ 2.16 ppm (quintet-like, *J* = 7.0 Hz) confirmed the above assignment (Fig. S4). The HMBC spectrum displayed a ^2^*J*_CH_ and a ^3^*J*_CH_ coupling between H8 and H9, respectively, and a quaternary, oxygen bearing carbon at δ 146.8 ppm which verified the C9–C10 connectivity between the alkenyl chain and the methyl furan moiety (Fig. S5). This feature unequivocally confirmed the double bond in conjugation with the β-methyl-substituted methyl furan moiety as indicated by GC/MS measurements [8]. Also, a ^3^*J*_CH_ long-range correlation of C10 with the methyl group at δ 1.97 ppm on the furan ring (C19) unambiguously established that both the alkenyl and methyl groups were located on the same side of the furan moiety, i.e. in α- and β-position. Furthermore, a ^2^*J*_CH_ correlation between the methyl group and a quaternary carbon at δ 116.4 ppm (C11) along with a ^3^*J*_CH_ to the hydrogen bearing C12 at δ 109.0 ppm (βʹ-position) established the substitution pattern of the furan moiety as shown in Fig. 6a. Further evaluation of the broadband homonuclear decoupled TOCSY (Fig. S6) and HMBC (Fig. S5) spectra enabled the unequivocal assignment of carbons C7 to C5 in the alkenyl chain (Fig. 6a). Since GC/MS data unequivocally verified the presence of a methoxy group attached on C1 (in form of a methyl ester), the missing ^1^H and ^13^C NMR shifts could be assigned starting from the head group. Specifically, ^1^H, ^1^H TOCSY, COSY, and ^1^H,^13^C super long-range HMBC correlations [42, 43, 45, 47–49] were used to display up to ^5^*J*_CH_ couplings. For instance, a ^4^*J*_CH_ long-range correlation between the methoxy group (C20) and the methylene protons at δ 2.31 ppm (C2) directly attached to the ester carbonyl C atom at δ 174.0 ppm (C1) unambiguously fixed a methyl ester moiety on C1 (Fig. S7).

The pentyl chain in αʹ-position of the furan moiety, clearly indicated by GC/MS, could be completely assigned by 1D selTOCSY (Fig. S8), COSY, HSQC without decoupling, and HMBC. Also, its linkage to the quaternary carbon C13 at δ 154.9 ppm could be verified via HMBC correlations of the furan carbons C12 and C13 and the methylene protons H14 of the pentyl chain at δ 2.55 ppm (bt, *J* = 7.7 Hz, Fig. 6a). Accordingly, the structure of the most prominent u9M5-ME isomer in the mixture could be unequivocally elucidated to be (*E*)-9-(3-methyl-5-pentylfuran-2-yl)non-8-enoic acid. Similarly to saturated FuFAs, the short-term was coined* E*-9:1M5-ME. This short-term implicates the premise that the additional double bond in the carboxyalkyl chain was in conjugation with the furan moiety, as unequivocally shown in this work.

In the same way, the structure of the second most abundant u9M5-ME isomer could be established to be (*E*)-9-(3-methyl-5-pent-1-enylfuran-2-yl)nonanoic acid (*E*-9M5:1-ME, peak 3, Fig. 4c, Fig. 6b). In brief, the two diagnostic olefinic protons H14 at 6.09 ppm (d, *J* = 15.9 Hz) and H15 at 6.04 ppm (dt, *J*_*1*_ = 6.8, *J*_*2*_ = 15.7) (Fig. 5b, signals #C and #D) proved the presence of a *trans-*or *(E)*-configured double bond. In contrast to the major compound *E*-9:1M5-ME (peak 4, Fig. 4c), the additional double bond was located in the five carbon-containing side chain in αʹ-position of the furan moiety which was attached vicinal to the aromatic furan proton H12 (Fig. 6b). HMBC correlations between H15 as well as H14 and C13 (δ 150.4 ppm) confirmed a double bond directly connected to C13 and therefore in conjugation with the furan moiety. Vice versa, the saturated carboxyalkyl chain was linked to C10 of the methyl furan moiety as shown by HMBC correlations of the methylene protons H9 at δ 2.52 ppm (t, *J* = 7.6 Hz) to carbon C10 at δ 150.4 ppm which was isochronic with C13 (Fig. 6b, Table 1).

**Table 1:** δ_C_ (ppm) and δ_H_ (ppm) chemical shift, spin multiplicity, *J* coupling constants (Hz), and important HMBC correlation of the two *E*-configured u9M5-ME isomers *E*-9:1M5-ME (GC/MS peak 4) and *E*-9M5:1-ME (GC/MS peak 3)

Atom	*E*-9:1M5-ME (peak 4)			*E*-9M5:1-ME (peak 3)		
	δ_C_^a^ (ppm), type	δ_H_ (ppm), multiplicity, *J* (Hz)	Important HMBC	δ_C_^a^ (ppm), type	δ_H_ (ppm), multiplicity, *J* (Hz)	Important HMBC
1	174.0, C	-		nd^c^, C	nd^c^	
2	34.0, CH_2_	2.31 t (7.4)	C1, C3, C4	nd^c^, CH_2_	nd^c^	
3	24.8, CH_2_	1.64^b^		nd^c^, CH_2_	nd^c^	
4	28.9, CH_2_	1.33^b^		nd^c^, CH_2_	nd^c^	
5	28.7, CH_2_	1.34^b^		nd^c^, CH_2_	nd^c^	
6	29.3, CH_2_	1.45 quintet-like (7.2)^d^		nd^c^, CH_2_	nd^c^	
7	32.9, CH_2_	2.16 q-like (7.0)	C5, C6, C8, C9	28.9, CH_2_	1.30^b^	
8	126.8, CH	5.97, dt (15.6, 7.0)	C6, C10	28.5, CH_2_	1.59^b^	
9	117.0, CH	6.15 br d (15.7)	C7, C10	25.4, CH_2_	2.52, t (7.6)	C10, C11
10	146.6, C	-		150.4, C	-	
11	116.4, C	-		115.0, C	-	
12	109.0, CH	5.80 s	C10, C11, C13, C19	109.4, CH	5.91 s	C10, C11, C13, C19
13	154.7, C	-		150.4, C	-	
14	28.1, CH_2_	2.55 br t (7.7)	C12, C13, C15, C16	118.8, CH	6.09 d (15.9)	C13, C16
15	27.7, CH_2_	1.61 quintet-like (7.7)^d^		128.0, CH	6.04 dt (6.8, 15.7)	C13, C16, C17
16	31.3, CH_2_	1.34^b^		34.7, CH_2_	2.12 q-like (7.3)^d^	
17	22.3, CH_2_	1.34 sextet-like (7.6)^d^		22.3, CH_2_	1.46 sextet-like (7.2)^d^	
18	13.9, CH_2_	0.89 br t (6.9)	C16, C17	13.5, CH_2_	0.93 t (7.3)	C16, C17
19	9.8, CH_3_	1.97 s	C10, C11, C12	9.6, CH_3_	1.90 s	C10, C11, C12
20	51.3, CH_3_	3.66 s		nd^c^, CH_3_	nd^c^	

^a 13^C chemical shifts were indirectly determined by HSQC and HMBC due to low sample amount. ^b ^Multiplet pattern and coupling constants could not be determined because of strong signal overlap. ^c ^Not determined (due to heavy overlap with other resonances, the chemical shifts and/or coupling constants of these signals could not be unambiguously assigned). ^d^ Coupling pattern and constants derived by selective 1D TOCSY and/or band-selective HSQC without decoupling. Coupling constants were directly taken from the spectra and are not averaged

The structure of the two low abundant u9M5-ME isomers (peaks 1 and 2, Fig. 4c) with potential *Z*-configured double bonds could not be fully determined by NMR. Yet, tentative indications for a *Z*-configurated double bond were found by means of a doublet at δ 6.02 ppm in the ^1^H NMR spectrum at very low abundance and further substantiated by the four signals at δ 115 ppm in the selective HSQC without decoupling (Fig. S9, blue circle).

### Elution pattern of uFuFA isomers in GC/MS

The abundance ratio of the two major u9M5-ME isomers (peak 4/peak 3 = 2) in the fully homoband decoupled ^1^H NMR spectrum based on PSYCHE (signals #A and #C, Fig. 5b) agreed reasonably well with the one determined by GC/MS (peak 4/peak 3 = 2.5). Hence, *E*-9:1M5-ME corresponded with GC/MS peak 4 (56%) and *E*-9M5:1-ME with GC/MS peak 3 (23%). Also, the NMR measurements verified the conjugation of the additional double bond, respectively, with the furan moiety as previously indicated by GC/MS data [10, 11, 13, 16]. Accordingly, the low abundant isomers (GC/MS peaks 1 and 2, abundances 12% and 9%, respectively) represented the two *Z*-configurated u9M5-ME isomers. Since the position of the double bond (carboxyalkyl or alkyl chain) could be unequivocally assigned by GC/MS via the ion formed by a McLafferty-like rearrangement [8, 13], the GC/MS elution order could be established to be *Z*-9:1M5-ME < *Z*-9M5:1-ME < *E*-9M5:1-ME < *E*-9:1M5-ME on the polar Rtx-2330 capillary column (section “Materials and methods”). Remarkably, the corresponding GC elution order had been predicted for four u9D5-ME isomers on an OV101 capillary column [13]. Virtually the same uFuFA-ME fingerprint (i.e., abundance pattern and ∆t_R_ between the four isomers) had also been observed for the four u11D5-ME, u11M5-ME, and u11D3-ME isomers, respectively, detected in enriched fish oils [8]. This reported elution pattern was also valid for the commonly used nonpolar HP-5 column (Fig. S10). Accordingly, E-uFuFA-ME isomers generally eluted much later than the corresponding Z-FuFA-ME isomers from all GC columns tested so far. Compared to conventional FAMEs, e.g., 18:1-ME isomers (Rtx-2330 column; 18:1*tr*-ME < 18:1-ME; Δt_R_ 0.17 min) [8], not only the *E*/*Z* elution order was reversed but also the difference in retention times (∆t_R_) between the geometric uFuFA-ME isomers was much higher. Specifically, Δt_R_ between *Z*-9M5:1-ME and *E*-9M5:1-ME was 0.96 min, and Δt_R_ between *Z*-9:1M5-ME and *E*-9:1M5-ME was even > 3 times higher (3.3 min; Fig. S10). On the polar Rtx-2330 column, the RRI based on 12:0-ME and 18:3*n*-3-ME for u9M5 isomers were between 1.25 and 1.41. This corresponded to the RRI of the conventional fatty acids EPA (1.26) and 24:1*n*-9-ME (1.40, Table S1 and S2).

The few reports in scientific literature indicated that uFuFAs are generally occurring in a set of four isomers with *E*-uFuFAs contributing ~ 75% and *Z*-uFuFA only ~ 25% (latex, fish oil; Table S4) [8, 13]. Hence, it is likely that studies which only reported the presence of two uFuFA isomers actually only detected the most abundant and later eluting *E*-uFuFA isomers. The low amounts together with the described special chromatographic behavior and the lack of mass spectrometric data of uFuFAs in the online available NIST database [41] aggravated the detection of the full array of four uFuFA isomers. In this context, GC/MS runs of Kirres et al. (2018) [12] stored in our laboratory’s security drive could be re-inspected with regard to the new knowledge. Data from 29 plant samples fully verified the presence of four uFuFA isomers in the aforementioned abundance ratios of *E*- and *Z*-uFuFAs (Table S4) [8].

Yet, the origins of uFuFAs and their formation are currently unknown. In addition to their possible biosynthetic formation, they could also be transformation products of saturated FuFAs since small amounts of uFuFAs were observed exemplarily in laboratory experiments with FuFAs in the presence of water [13] or after the aqueous homogenization of beef liver [16]. Still, the largely constant fingerprints, regardless of the substance under consideration, were remarkable in this context (Table S4). Also, the function of the double bond is not clear at the moment. The additional conjugated double bond of uFuFAs could increase the stability of the molecule by resonance stabilization compared to saturated FuFAs. If and to what extent this may have an impact on the antioxidant properties is currently unknown. These open questions produce evidence that more research is necessary in this field and the presented determination of the configuration of the double bond will be helpful for such investigations.

## Conclusion

The combination of CCC and (silver ion) column chromatography enabled the removal of 99.76% of the weight of the methylated latex glove extract. Specifically, the high sample capacity of CCC (1 g) used in the first step removed the bulk of the matrix. This enrichment of the analytes makes it possible to measure sample extracts in a more concentrated form. Unfortunately, such (GC/MS) measurements frequently indicate the presence of co-eluting compounds that could not be detected and thus are not predictable before the CCC fractionation [50, 51]. This intrinsic problem gets the more relevant the smaller the amount of the analyte in the sample is. In the present case, 9M3-ME (same ECL value as the target u9M5 isomers), 9D3-ME (Fig. 3), and further, unidentified compounds (Fig. 4b, peaks marked with an asterisk) reduced the purity of CCC fractions containing the target u9M5-ME isomers. Since these impurities had the same or very similar *K* values as the analytes, they are difficult to remove by CCC. Instead, complementary techniques have to be applied like (silver ion) column chromatography in the present case. However, the effective removal of the sample matrix by CCC made it possible that the remaining sample could be directly applied to subsequent steps with a lower capacity. This approach provided ~ 2.4 mg of the uFuFA-ME isomer mixture, which met the purity requirement for the application of sophisticated NMR methods which were recently made available. In this mixture, doublets and multiplets resulting from proton–proton coupling of the all four u9M5-ME isomers at very similar chemical shifts made it virtually impossible to make correct assignments in 1D and 2D NMR experiments. This problem could be solved by removal of all proton–proton couplings by PSYCHE [44, 45]. The subsequent application of further 1D and 2D NMR methods enabled an unequivocal structure determination of the most prominent isomers in the isomeric mixture to be *E*-u9:1M5-ME and *E*-u9M5:1-ME. Moreover, linking NMR with GC/MS data enabled the assignment of the four uFuFA isomers in the GC chromatogram. Specifically, GC retention increased in the order *Z*-9:1M5-ME < *Z*-9M5:1-ME < *E*-9M5:1-ME < *E*-9:1M5-ME both on a highly polar Rtx-2330 column and a nonpolar HP5-MS column. Previously we found that the peak pattern (abundance ratios and differences in GC retention times) was also valid for other uFuFA isomer groups [8]. Hence, the sophisticated work applied in this study can be omitted in future in the case of other uFuFA isomers.

### Supplementary information

Below is the link to the electronic supplementary material.

Supplementary file1 (PDF 734 KB)

## Data Availability

The datasets created and analyzed in this study are available upon reasonable request from the corresponding author.
