# Supplementary material for: Enrichment and structural assignment of geometric isomers of unsaturated furan fatty acids
Source: Anal Bioanal Chem. 2023 Aug 21;415(25):6333–43. doi: 10.1007/s00216-023-04908-z (PMC10558370; doi:10.1007/s00216-023-04908-z)
Supplement: Supplementary file 1 — Supplementary file1 (PDF 734 KB) [file 216_2023_4908_MOESM1_ESM.pdf]

Supplementary Information for

Enrichment and structural assignment of geometric isomers of unsaturated furan fatty acids

Franziska Müller[1], Jürgen Conrad[2], Tim Hammerschick[1], and Walter Vetter[1*]

[1] Department of Food Chemistry (170b), Institute of Food Chemistry, University of Hohenheim, Garbenstr. 28, 70599 Stuttgart, Germany

[2] Department of Bioorganic Chemistry (130b), Institute of Chemistry, Hohenheim University, Garbenstr. 30, 70599 Stuttgart, Germany

***corresponding Author**
Walter Vetter
Department of Food Chemistry (170b)
Institute of Food Chemistry
University of Hohenheim
Garbenstr. 28
D-70593 Stuttgart, Germany
Walter.vetter@uni-hohenheim.de

Content

Figures ... 3

Fig. S1 GC/MS spectra of u9M5-ME isomers ... 3

Fig. S2 CCC elution profile of the latex extract ... 4

Fig. S3 GC/MS chromatograms of silica subfractions ... 5

Fig. S4a COSY spectrum (600 MHz, $CDCl_3$) ... 6

Fig. S4b F1-homoband decoupled CLIP COSY spectrum (600 MHz, $CDCl_3$) ... 6

Fig. S5 HMBC spectrum (600 MHz, $CDCl_3$) ... 7

Fig. S6 F1-homoband decoupled TOCSY spectrum (600 MHz, $CDCl_3$) ... 8

Fig. S7 Super long range HMBC spectrum (600 MHz, $CDCl_3$) ... 9

Fig. S8 1D selTOCSY of H-14 at δ 2.55 (600 MHz, $CDCl_3$) ... 9

Fig. S9 Excerpt of the selective HSQC spectrum (600 MHz, $CDCl_3$) ... 10

Fig. S10 Comparison of the separation on an Rtx-2330 and an HP-5 column ... 11

Tables ... 12

Table S1 Composition of the 37 component standard ... 12

Table S2 Characteristic *m/z* values, RRI and K values of detected FuFAs and uFuFAs ... 14

Table S3 GC/MS data of u9M5-ME isomers ... 15

Table S4 Share of *Z*- or *E*-uFuFAs in different samples. ... 15

Parameter of NMR analyses ... 16

References ... 18

Figures

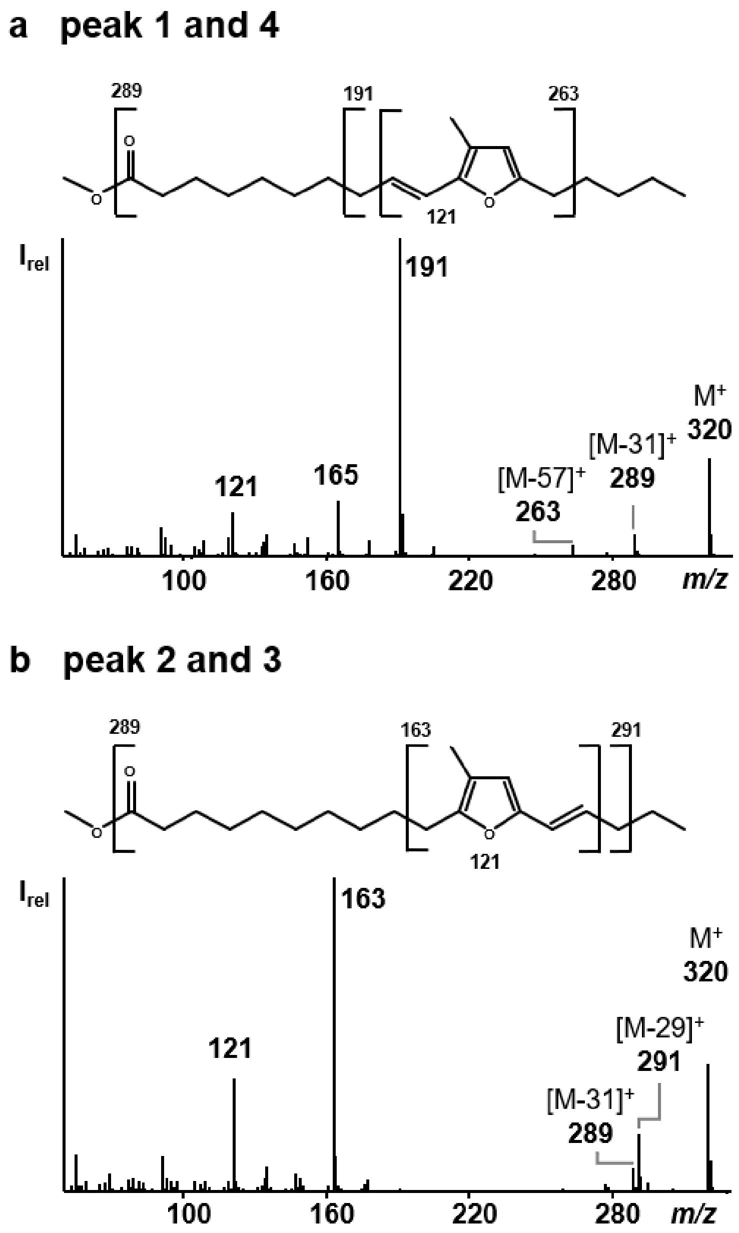

Fig. S1 GC/MS spectra of u9M5-ME isomers with inserted chemical structure of the *E*-isomers and marked fragmentation of (**A**) 9:1M5-ME (peak 1 and 4 according to Fig. 2), (**B**) 9M5:1-ME (peak 2 and 3 according to Fig. 2).

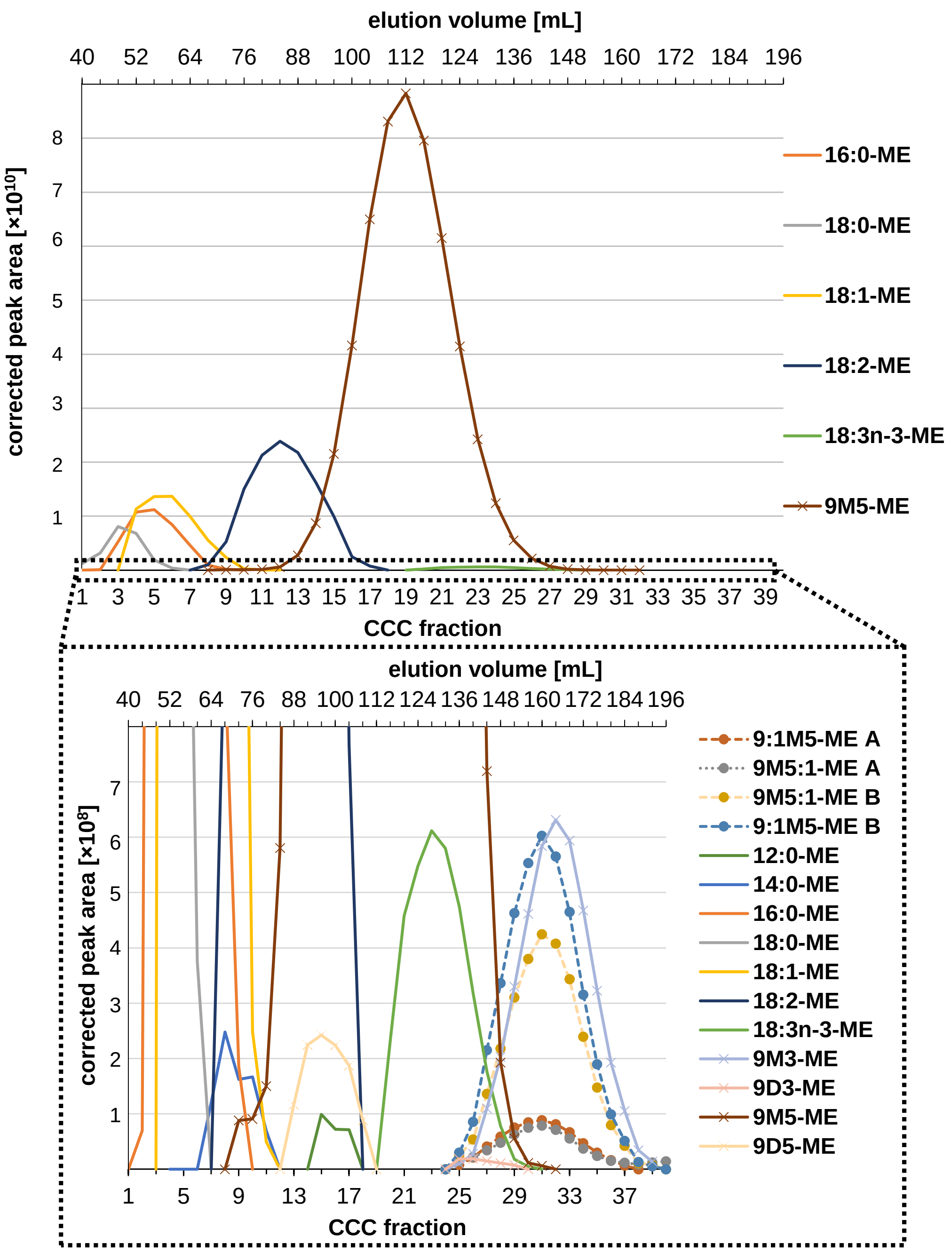

Fig. S2 Elution profile of the latex extract: CCC fractions were measured by GC/MS and the individual compounds were identified by their mass spectra and the retention times in comparison to a 37 compound FAME mix (**Table S1**) and a 9M5-ME and a 9D5-ME reference standard. The peak area was determined from the GC/MS chromatogram*, corrected via the internal standard 14:0-EE and the dilution factor. The data was smoothed by a weighted 9-point smoothing according Müller *et al.* (2019) [1] and plotted against the fraction number and the elution volume. *Due to a coelution of 9M5:1-ME A with a unknown substance in GC/MS in CCC fractions 25-29, only the base peak at *m/z* 163 was integrated and was corrected with the response factor F_R determined for 9M5:1 B (in the GC/MS chromatogram of fraction CCC_{25}) with $F_R = \frac{A_{TIC}}{A_{163}}$, $A_{TIC} = F_R * A_{163}$, A_{TIC} area of the total ion current (TIC) and A_{163} area of the ion trace *m/z* 163.

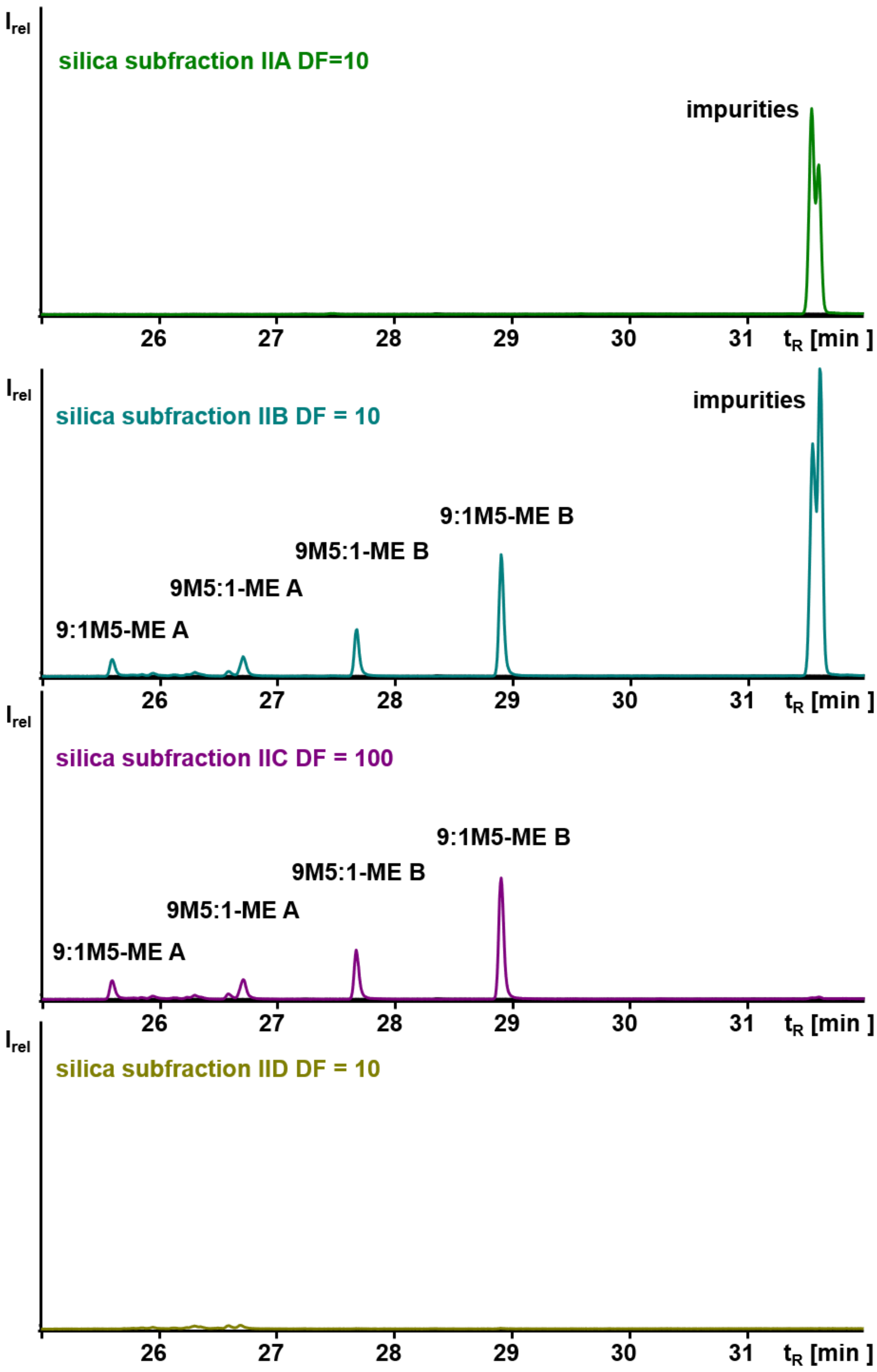

Fig. S3 GC/MS chromatograms of the four silica subfractions IIA-D with the used dilution factor (DF).

a

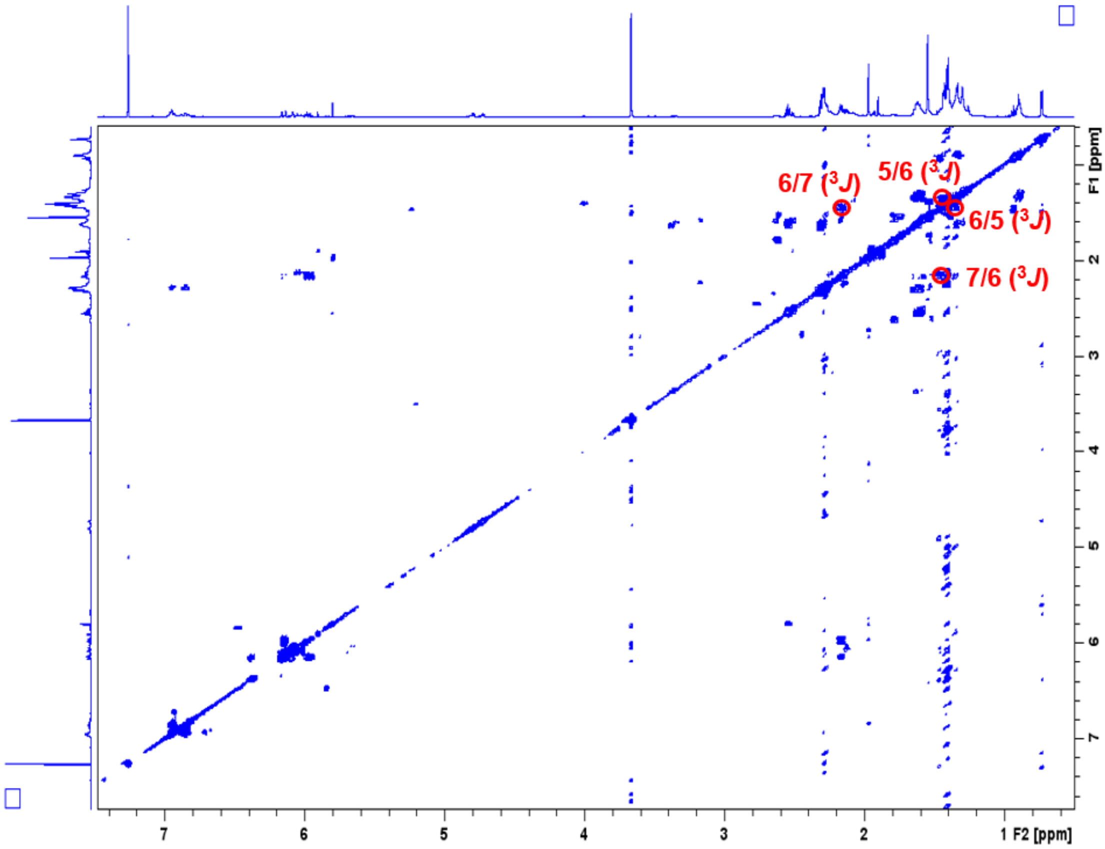

b

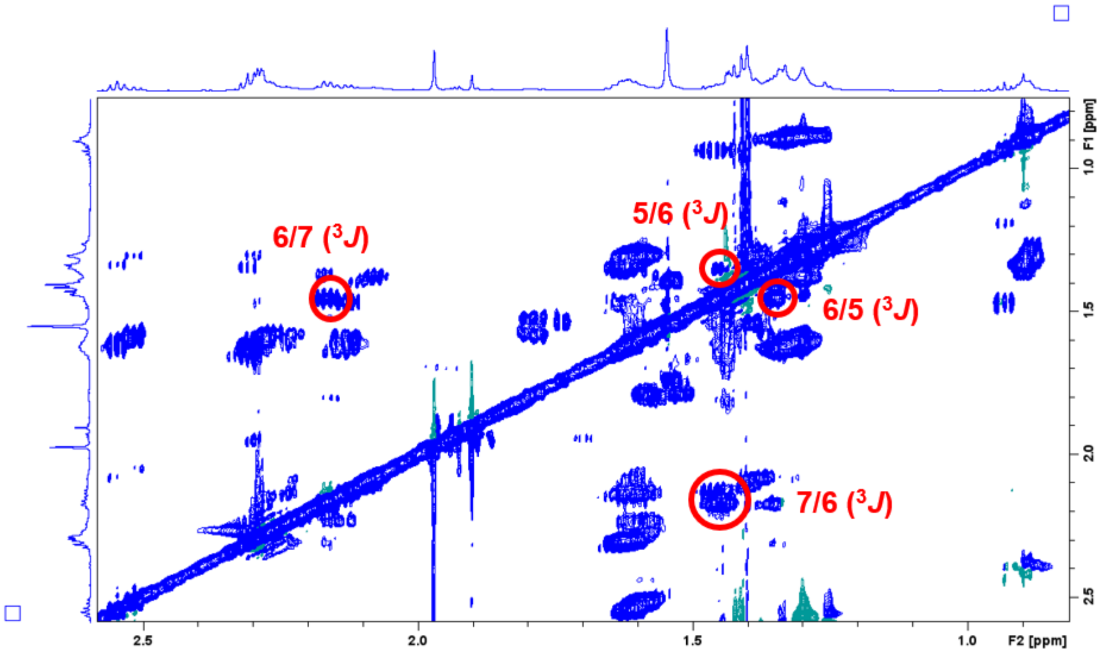

Fig. S4: Conventional COSY spectrum (600 MHz, $CDCl_3$) (**a**) of silica fraction IIC and expansion of F1-homoband decoupled CLIP COSY spectrum (600 MHz, $CDCl_3$) (**b**).

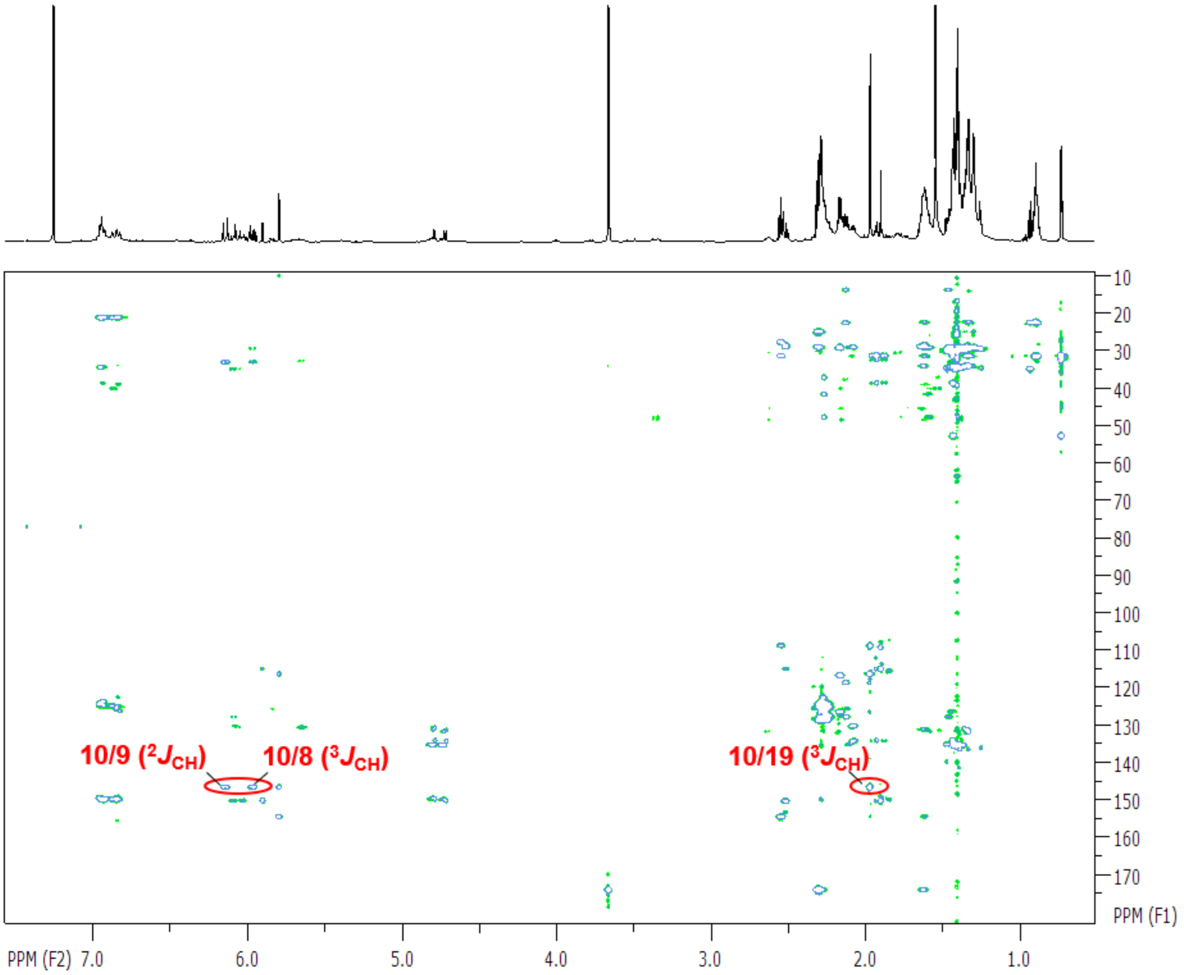

Fig. S5 HMBC spectrum (600 MHz, $CDCl_3$) of silica fraction IIC

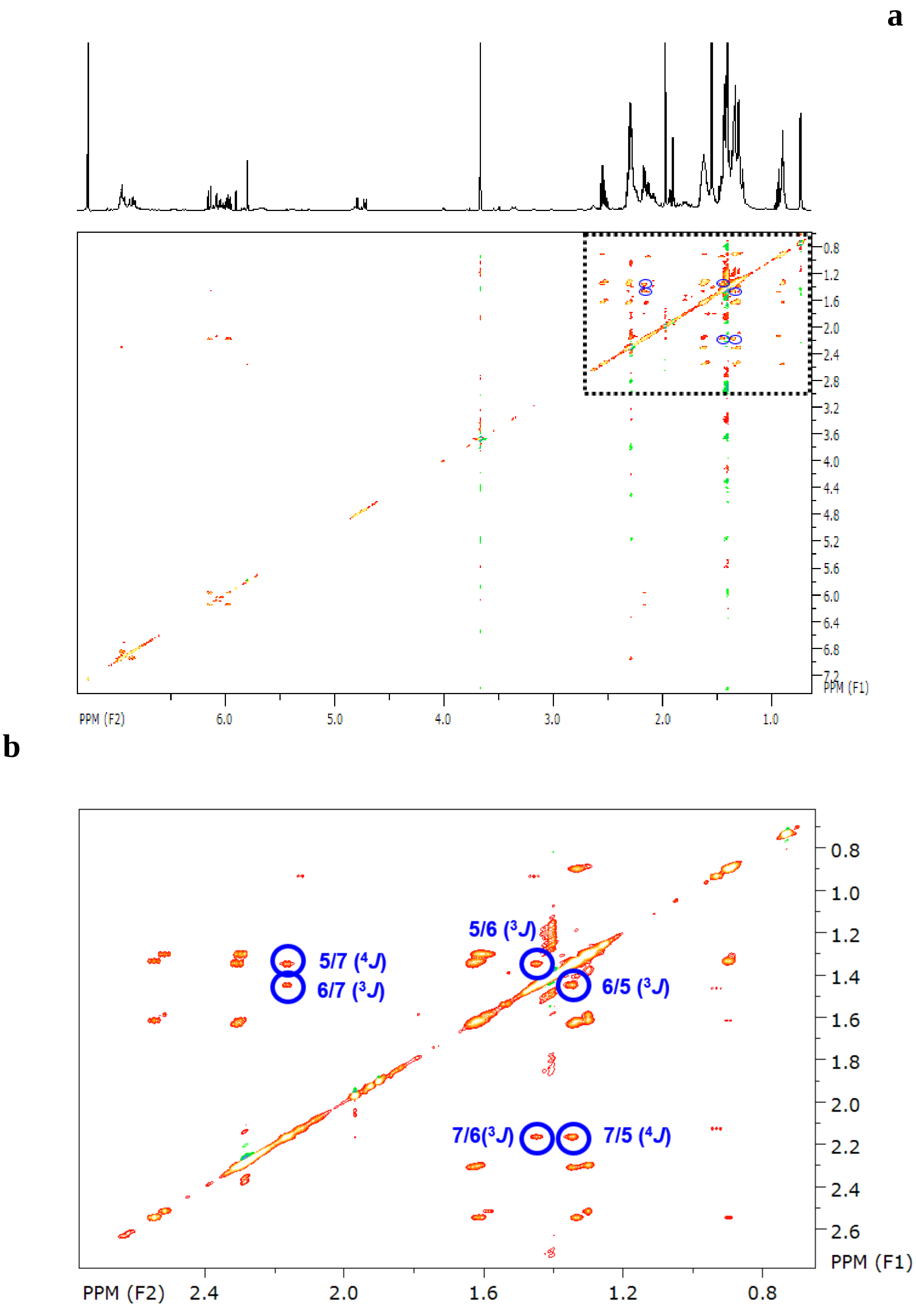

Fig. S6 F1-homoband decoupled TOCSY spectrum (600 MHz, $CDCl_3$) (**a**) and its expansion (**b**).

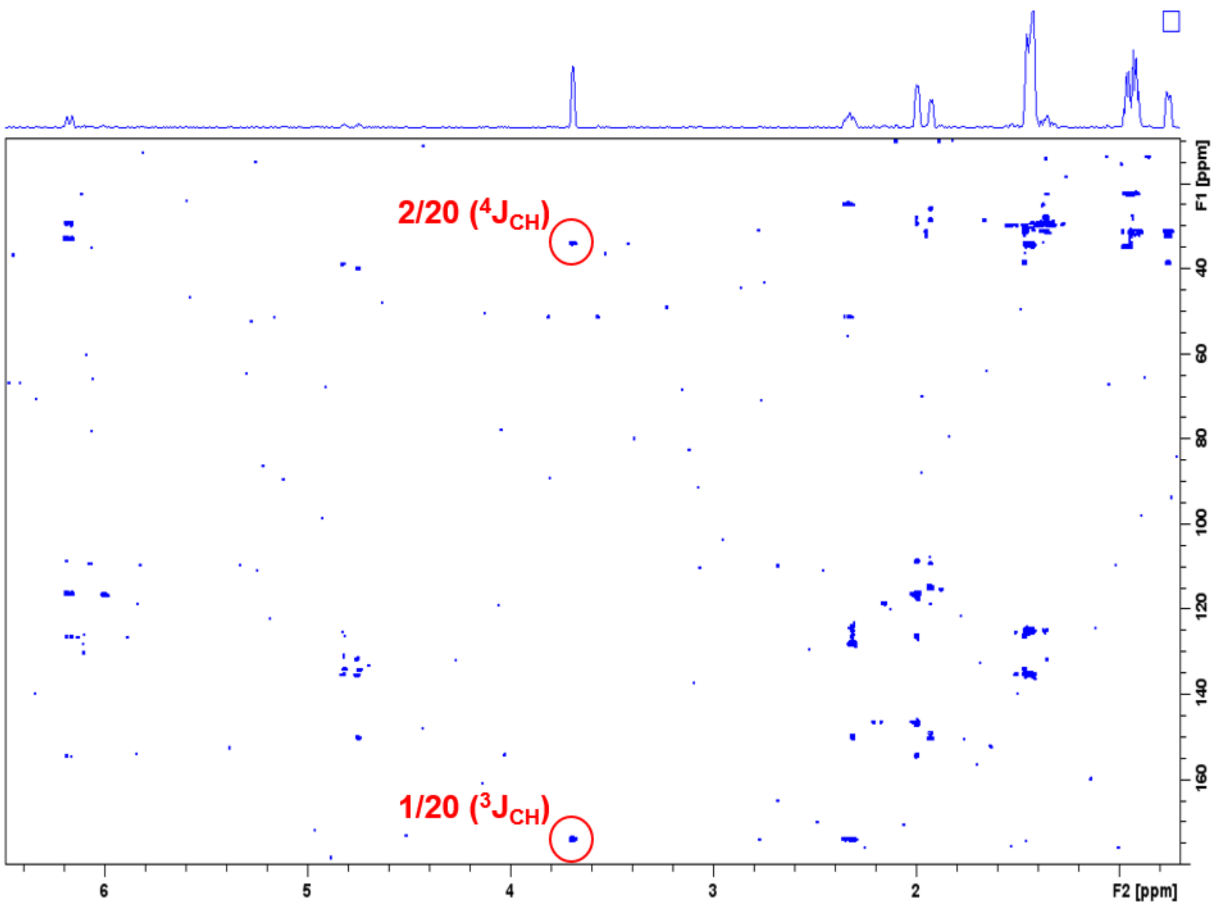

Fig. S7 Super long range HMBC spectrum (600 MHz, $CDCl_3$) of silica fraction IIC with 50% NUS sampling.

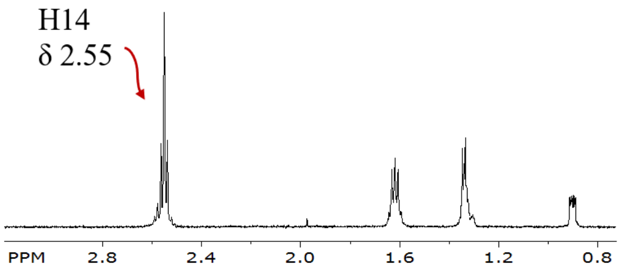

Fig. S8 1D selTOCSY (600 MHz, $CDCl_3$) of H-14 at δ 2.55 (indicated by arrow) of the major compound (*E*)-9:1M5-ME.

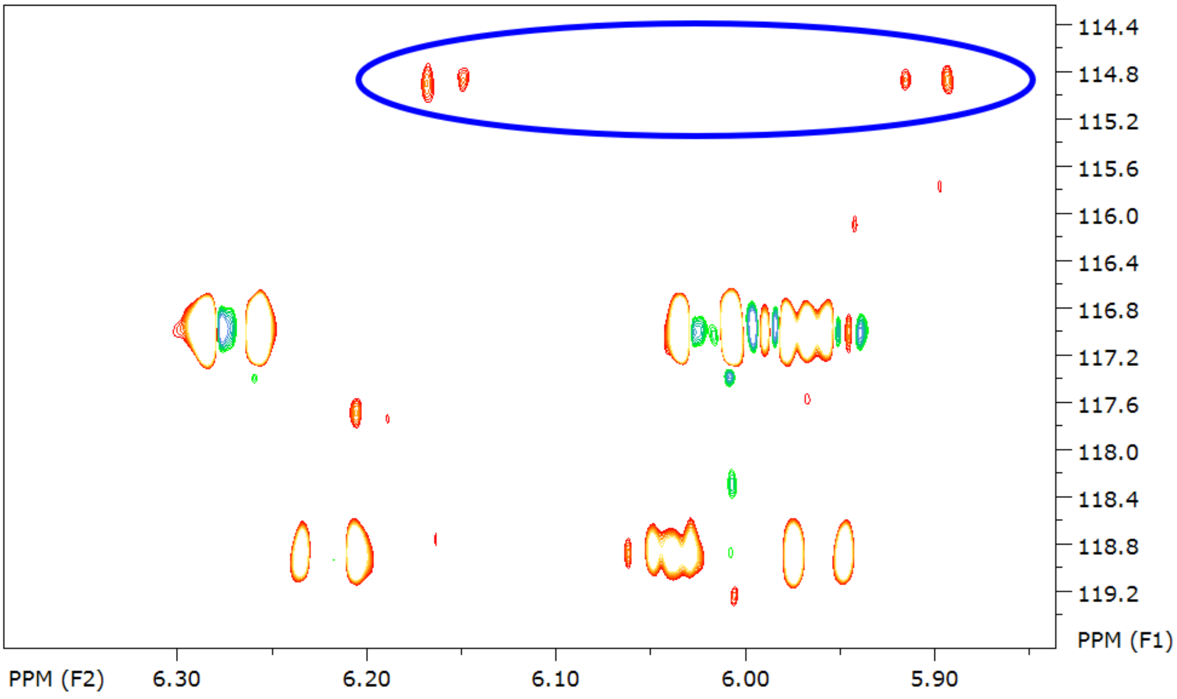

Fig. S9 Excerpt of the selective HSQC measured without decoupling (600 MHz, $CDCl_3$).

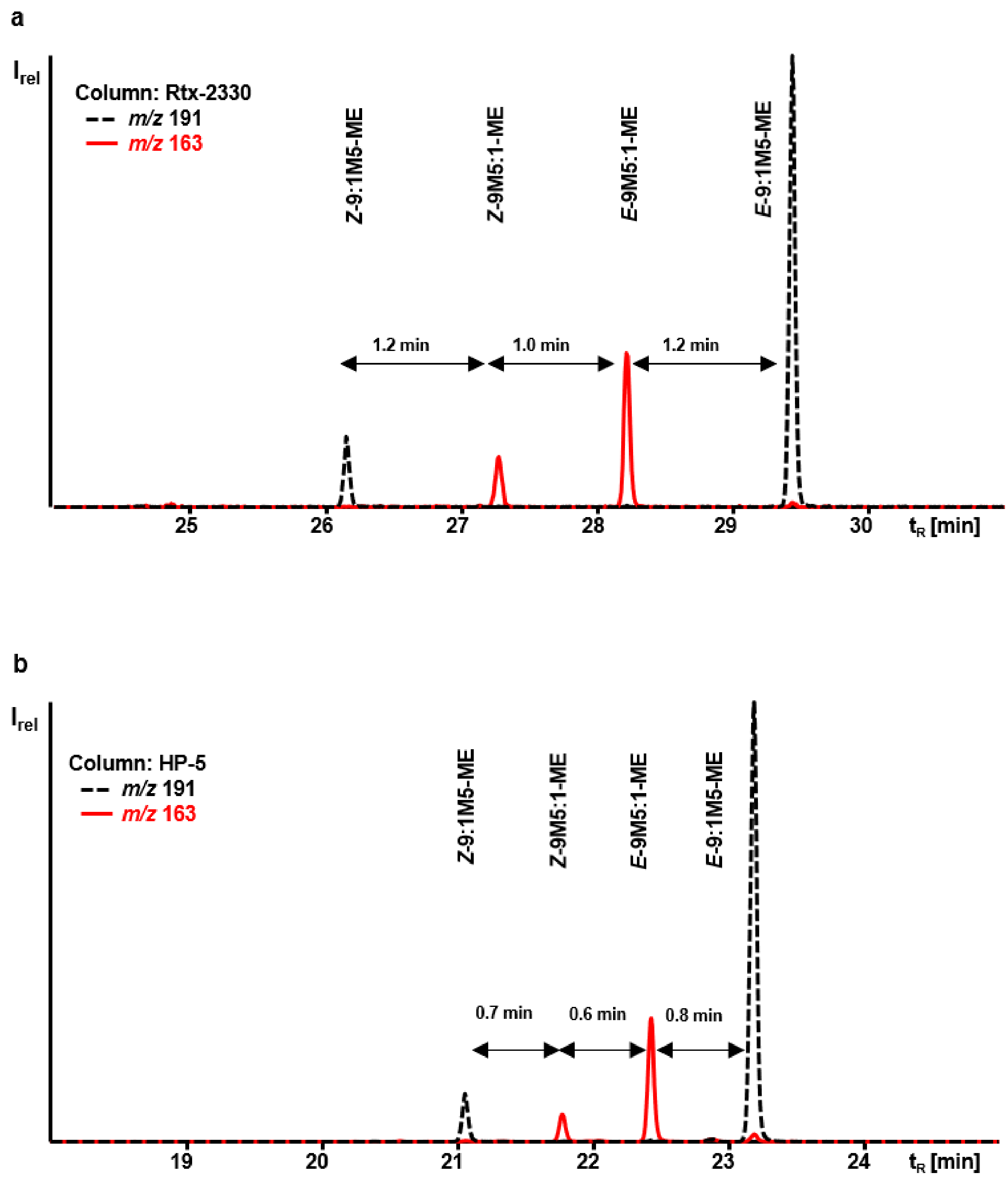

Fig. S10 Excerpts of GC/MS chromatograms (single ion track, *m/z* 163 and *m/z* 191) measured (**a**) on a polar Rtx-2330 column (section General Experimental Procedures) and (**b**) on a non-polar HP-5 column. The data for the HP-5 column were measured on the same GC/MS system described in the General Experimental Procedures section, a HP5-5MS UI, 5% phenyl, 95% methyl polysiloxane column (30 m × 0.25 mm internal diameter × 0.25 µm film thickness, Agilent, Waldbronn, Germany) was used and the settings were according to Wendlinger *et al.* (2016) [2]. The measurements were performed in the scan modus (*m/z* 50-550) after a solvent delay of 7 min.

Tables

Table S1 Composition of the 37 compound FAME standard (Sigma Aldrich) with their relative retention time RRI based on 12:0-ME and 18:3*n*-3-ME, K values calculated according to Müller *et al.* (2022) [3, 4], molecular ions $[M]^+$, and diagnostic ions. FAMEs detected in the CCC fractions of the disposable latex glove sample are marked in bold.

**FAME**	**RRI[a]**	**K value[b]**	**molecular ion $[M]^+$ *m/z***	**diagnostic ions[c] *m/z***	**diagnostic ion PUFA-ME[d] *m/z***	**concentration [µg/mL]**
4:0-ME	–[e]		102	74>87		4
6:0-ME	–[e]		130	74>87		4
8:0-ME	–[e]		158	74>87		4
10:0-ME	0.41		186	74>87		4
11:0-ME	0.45		200	74>87		4
**12:0-ME**	0.50	**0.35**	**214**	**74>87**		4
13:0-ME	0.54		228	74>87		4
**14:0-ME**	0.59	**0.19**	**242**	**74>87**		4
14:1*n*-5-ME	0.62		240	74>87>81>79		2
15:0-ME	0.64		256	74>87		**2**
15:1*n*-7-ME	0.68		**254**	74>87>81>79		**2**
**16:0-ME**	0.70	**0.14**	**270**	**74>87**		**6**
16:1*n*-7-ME	0.73		**268**	74>87>81>79		**2**
17:0-ME	0.77		**284**	74>87		**2**
17:1*n*-7-ME	0.81		**282**	74>87>81>79		**2**
**18:0-ME**	0.84	**0.09**	**298**	**74>87**		**4**
18:1*n*-9*tr*-ME	0.87		**296**	74>87>81>79		**2**
**18:1*n*-9-ME**	0.88	**0.16**	**296**	**74>87>81>79**		**4**
18:2*n*-6*tr*-ME	0.91		**294**	81>79		**2**
**18:2*n*-6-ME**	0.93	**0.27**	**294**	**81>79**		**2**
18:3*n*-6-ME	0.97		292	79>81	150>108	**2**
**18:3*n*-3-ME**	1.00	**0.49**	**292**	**79>81**	**108>>150**	**2**
20:0-ME	1.01		**326**	74>87		**4**
20:1*n*-9-ME	1.04		324	74>87>81>79		2
21:0-ME	1.10		340	74>87		2
**FAME**	**RRI[a]**	**K[b]**	**$[M]^+$**	**ions[c]**	**ions[d]**	**concentration**

Table 1 continued

FAME	RRI[a]	K value[b]	molecular ion $[M]^+$ *m/z*	diagnostic ions[c] *m/z*	diagnostic ion PUFA-ME[d] *m/z*	concentration [µg/mL]
20:2*n*-6-ME	1.10		322	81>79		2
20:3*n*-6-ME	1.14		320	79>81	150>108	2
20:3*n*-3-ME	1.18		320	79>81	108>>150	2
20:4*n*-6-ME	1.18		318	79>81	150>108	2
22:0-ME	1.19		354	74>87		4
22:1*n*-9-ME	1.22		352	74>87>81>79		2
20:5*n*-3-ME	1.26		316	79>81	108>>150	2
23:0-ME	1.28		368	74>87		2
22:2*n*-6-ME	1.28		350	81>79		2
24:0-ME	1.37		382	74>87		4
22:6*n*-3-ME	1.47		342	79>81	108>>150	2
24:1*n*-9-ME	1.40		380	74>87>81>79		2

[a] relative retention time index (RRI) on basis of 12:0-ME and 18:3*n*-3-ME: $RRI = \frac{t_{R,Analyt}}{(t_{R,12:0-ME}+t_{R,18:3n-3-ME})} \cdot 1.5$;
[b] partition coefficient calculated from the fraction data of the CCC fraction with the highest abundance for each substance according Müller *et al.* (2023): $K = \frac{V_E - V_M}{(V_C - V_M)}$ with V_M volume mobile phase, V_E elution volume and V_C total column capacity [3, 4].
[c] diagnostic ions to distinguish between saturated, monoen, dien and polyen FAME [5]; [d] the position of the last double bond in polyunsaturated fatty acid methyl ester (PUFA-ME) (*n*-3 or *n*-6) can be determined for PUFA with methylene interrupted double bonds with the help of *m/z* 108 or *m/z* 150 [6]; [e] not determined in the used method.

Table S2 GC/MS data of all identified FuFA and uFuFA isomers in the lipid extract of disposable latex gloves: Relative retention time RRI based on 12:0-ME and 18:3*n*-3-ME, K values calculated according to Müller *et al.* (2022) [3, 4]; molecular ions, McLafferty ion (distinguish between D-/M-FuFA), base peak, the $[M-31]^+$ fragment (α-cleavage of the methoxy group; indicating the methyl ester) and the allylic cleavage in the alkyl chain.

**FuFA-ME**[a]	**RRI**[b]	**K value**[c]	**molecular ion $[M]^+$ *m/z***	**McLafferty ion *m/z***	**base peak *m/z***	**$[M-31]^+$ *m/z***	**allylic cleavage**[d] ***m/z***
9M3-ME	0.97	0.66	294	109	137	263	265 ($[M-29]^+$)
9D3-ME	1.03	0.54	308	123	151	277	279 ($[M-29]^+$)
9M5-ME	1.11	0.41	322	109	165	291	265 ($[M-57]^+$)
9D5-ME	1.17	0.33	336	123	179	305	279 ($[M-57]^+$)
9:1M5-ME (1)	1.25	0.64	320	121	191	289	263 ($[M-57]^+$)
9M5:1-ME (2)	1.30	0.64	320	121	163	289	291 ($[M-29]^+$)
9M5:1-ME (3)	1.35	0.64	320	121	163	289	291 ($[M-29]^+$)
9:1M5-ME (4)	1.41	0.64	320	121	191	289	263 ($[M-57]^+$)

[a] short hand names according Vetter *et al.* (2012) [7] and Kirres and Vetter (2016) [8], u9M5 isomers are numbered according to the peak numbering in the manuscript Figure 2; [b] relative retention time index (RRI) on basis of 12:0-ME and 18:3*n*-3-ME: $RRI = \frac{t_{R,Analyt}}{(t_{R,12:0-ME} + t_{R,18:3n-3-ME})} \cdot 1.5$; [c] partition coefficient calculated from the fraction data of the CCC fraction with the highest abundance for each substance according Müller *et al.* (2023): $K = \frac{V_E - V_M}{(V_C - V_M)}$ with V_M volume mobile phase, V_E elution volume and V_C total column capacity [3, 4]; [d] *m/z* of the allylic cleavage in the alkyl chain; the resulting alkyl loss is given in brackets

Table S3 characteristic mass to charge (*m/z*) ratios and the relative abundances (I_{rel}) of these GC/MS (fragment) ions of the four investigated u9M5-ME isomers.

GC/MS (fragment) ions	9:1M5-ME (1) *m/z* (I_{rel} [%])	9M5:1-ME (2) *m/z* (I_{rel} [%])	9M5:1-ME (3) *m/z* (I_{rel} [%])	9:1M5-ME (4) *m/z* (I_{rel} [%])
$[M]^+$	320 (33)	320 (47)	320 (50)	320 (36)
$[M-31]^+$	289 (8)	289 (9)	289 (9)	289 (8)
$[M-29]^+$	–	291 (18)	291 (18)	–
$[M-57]^+$	263 (4)	–	–	263 (4)
base peak	191 (100)	163 (100)	163 (100)	191 (100)
McLafferty-like ion	121 (13)	121 (32)	121 (28)	121 (12)
share in mixture [%]	9	12	23	56

Table S4 Share of *Z*- or *E*-uFuFAs in different samples.

uFuFA	n[a]	sample(s)	∑*Z*-uFuFAs	∑*E*-uFuFAs	References
u9M5	1	latex	21	79	this study
u9M5	1	latex	33	67	[9]
u11M5	1	fish oil[b]	34	66	[9]
u9D5	1	laboratory experiment	23[c]	77[c]	[10]
u11D3	1	fish oil[d]	27	73	[9]
u11D5	1	fish oil[b]	27	73	[9]
u11D5[e]	29	herbs, spinach, grass	21±6[f]	79±6[f]	[8]
u11D5	7	leafy vegetable, grass and herbs	24±5[f]	76±5[f]	unpublished data[g]

[a] sample number; [b] fish oil enriched in docosahexaenoic acid (DHA) [9]; [c] calculated from peak height of the published chromatogram [10]; [d] fish oil enriched in eicosapentaenoic acid (EPA) [9]; [e] re-evaluation of the chromatograms of Kirres *et al.* (2016) [8] stored in our lab, the originally published data reported only the occurrence of *E*-FuFAs; [f] mean value with standard deviation of all samples; [g] samples were extracted and enriched according to Kirres *et al.* (2016) [8] and measured as described by Müller *et al.* (2022) [9]

Parameter of NMR analyses

1D TSE-PSYCHE: p1 12.6 µs corresponding to a flip angle of 90°, relaxation delay d1 1 s, acquisition time aq 0.5 s, flip angle for PSYCHE pulse element 20°, band width of each CHIRP pulse bw 10 kHz, duration of double CHIRP PSYCHE element 30 ms, weak gradient for PSYCHE 1%, RF amplitude of first and second CHIRP frequency-swept pulses 446, duration of first and second CHIRP frequency-swept pulses 40 ms, weak gradient for first and second CHIRP swept-frequency 1%, TD 5000 (F2, sw 5000 Hz) and 24 (F1, sw 40 Hz), number of scans 32, the macro 'pshift' was used to process the data.

F1 homoband decoupled PSYCHE TOCSY: p1 12.6 µs (90°), d1 1.4 s, mixing time 70 ms, aq 0.22s, parameters of CHIRP frequency -swept pulses, PSYCHE pulse element and weak gradients were used as described above, TD 2048 (F2, sw 4690 Hz) and 1024 (F1, sw 4690 Hz), ns 16.

F1 homoband decoupled CLIP COSY: p1 12.6 µs (90°), d1 1.2 s, CLIP delay 12.5 ms, a psyche-double 10 kHz, 2 x15 ms PSYCHE pulse element with a total duration time of 32 ms and a weak gradient of 1% was used, smoothed CHIRP pulses bw 40 kHz and duration 5 ms, TD 4096 (F2, sw 4690 Hz) and 512 (F1, sw 4690 Hz), ns 20.

1D selective TOCSY: p1 12.6 µs (90°), d1 2.0 s, mixing time 100 ms, aq 2.7 s, Gaus180r pulses were used for selective excitation of resonances of interest, ns 128.

Gradient COSY (standard parameters): p1 12.6 µs (90°), d1 2.0 s, aq 0.18 s, 50 % NUS (non-uniform sampling in F1), TD 2048 (F2, sw 5681 Hz) and 512 (F1, sw 5681 Hz), ns 8.

Gradient HSQC (standard parameters): p1 12.6 µs (90°), d1 1.5 s, aq 0.17 s, TD 2048 (F2, sw 5868 Hz) and 128 (F1, sw 24901 Hz), ns 2.

Bandselective HSQC without decoupling: p1 12.6 µs (90°), d1 1.5 s, aq 0.35 s, TD 4096 (F2, sw 5868 Hz) and 256 (F1, sw 6301 Hz, offset 120 ppm), ns 64.

Gradient HMBC (standard parameters): p1 12.6 µs (90°), d1 1.5 s, aq 0.20 s, TD 2048 (F2, sw 5013 Hz) and 512 (F1, sw 30184 Hz), optimized for long range J_{CH} = 8 Hz, ns 64.

Super long range HMBC: p1 12.6 µs (90°), d1 1.0 s, aq 0.23 s, TD 2048 (F2, sw 5013 Hz) and 512 (F1, sw 30184 Hz), optimized for long range J_{CH} = 2 Hz, 50 % NUS, ns 64.

References

1. Müller M, Murić M, Glanz L, Vetter W. Improving the resolution of overlapping peaks by heartcut two-dimensional countercurrent chromatography with the same solvent system in both dimensions. J. Chromatogr. A. 2019; https://doi.org/10.1016/j.chroma.2019.03.012
2. Wendlinger C, Hammann S, Vetter W. Detailed study of furan fatty acids in total lipids and the cholesteryl ester fraction of fish liver. Food Anal. Methods. 2016; https://doi.org/10.1007/s12161-015-0211-x
3. Ito Y. Golden rules and pitfalls in selecting optimum conditions for high-speed counter-current chromatography. J. Chromatogr. A. 2005; https://doi.org/10.1016/j.chroma.2004.12.044
4. Müller F, Hermann-Ene V, Schmidpeter I, Hammerschick T, Vetter W. Furan fatty acids in some 20 fungi species: unique profiles and quantities. J. Agric. Food Chem. 2022; https://doi.org/10.1021/acs.jafc.2c05100
5. Thurnhofer S, Vetter W. A gas chromatography/electron ionization-mass spectrometry-selected ion monitoring method for determining the fatty acid pattern in food after formation of fatty acid methyl esters. J. Agric. Food Chem. 2005; https://doi.org/10.1021/jf051468u
6. Fellenberg AJ, Johnson DW, Poulos A, Sharp P. Simple mass spectrometric differentiation of the *n*-3, *n*-6 and *n*-9 series of methylene interrupted polyenoic acids. Biol. Mass Spectrom. 1987; https://doi.org/10.1002/bms.1200140306
7. Vetter W, Laure S, Wendlinger C, Mattes A, Smith AWT, Knight DW. Determination of furan fatty acids in food samples. J Am Oil Chem Soc. 2012; https://doi.org/10.1007/s11746-012-2038-6
8. Kirres C, Vetter W. Furan fatty acid content and homologue patterns of fresh green matrices. J. Food Compos. Anal. 2018; https://doi.org/10.1016/j.jfca.2018.01.001
9. Müller F, Hammerschick T, Vetter W. Geometrical and positional isomers of unsaturated furan fatty acids in food. Lipids. 2023; https://doi.org/10.1002/lipd.12364
10. Jandke J, Schmidt J, Spiteller G. Über das Verhalten von F-Säuren bei Oxidation mit Lipoxydase in Anwesenheit von SH-haltigen Verbindungen. Liebigs Ann. Chem. 1988; https://doi.org/10.1002/jlac.198819880107
